# Treatment With Suboptimal Dose of Benznidazole Mitigates Immune Response Molecular Pathways in Mice With Chronic Chagas Cardiomyopathy

**DOI:** 10.3389/fcimb.2021.692655

**Published:** 2021-07-14

**Authors:** Priscila Silva Grijó Farani, Khodeza Begum, Glaucia Vilar-Pereira, Isabela Resende Pereira, Igor C. Almeida, Sourav Roy, Joseli Lannes-Vieira, Otacilio Cruz Moreira

**Affiliations:** ^1^ Real Time PCR Platform RPT09A, Laboratory of Molecular Biology and Endemic Diseases, Oswaldo Cruz Institute, Oswaldo Cruz Foundation, Rio de Janeiro, Brazil; ^2^ Laboratory of Biology of the Interactions, Oswaldo Cruz Institute, Oswaldo Cruz Foundation, Rio de Janeiro, Brazil; ^3^ Department of Biological Sciences, Border Biomedical Research Center, University of Texas at El Paso, El Paso, TX, United States

**Keywords:** Chagas disease, *Trypanosoma cruzi*, cardiomyopathy, immune response, TaqMan array, benznidazole, pentoxifylline

## Abstract

Chronic Chagas cardiomyopathy (CCC) is the most frequent and severe form of Chagas disease, a neglected tropical illness caused by the protozoan *Trypanosoma cruzi*, and the main cause of morbimortality from cardiovascular problems in endemic areas. Although efforts have been made to understand the signaling pathways and molecular mechanisms underlying CCC, the immunological signaling pathways regulated by the etiological treatment with benznidazole (Bz) has not been reported. In experimental CCC, Bz combined with the hemorheological and immunoregulatory agent pentoxifylline (PTX) has beneficial effects on CCC. To explore the molecular mechanisms of Bz or Bz+PTX therapeutic strategies, C57BL/6 mice chronically infected with the *T. cruzi* Colombian strain (discrete typing unit TcI) and showing electrocardiographic abnormalities were submitted to suboptimal dose of Bz or Bz+PTX from 120 to 150 days postinfection. Electrocardiographic alterations, such as prolonged corrected QT interval and heart parasite load, were beneficially impacted by Bz and Bz+PTX. RT-qPCR TaqMan array was used to evaluate the expression of 92 genes related to the immune response in RNA extracted from heart tissues. In comparison with non-infected mice, 30 genes were upregulated, and 31 were downregulated in infected mice. Particularly, infection upregulated the cytokines IFN-γ, IL-12b, and IL-2 (126-, 44-, and 18-fold change, respectively) and the T-cell chemoattractants CCL3 and CCL5 (23- and 16-fold change, respectively). Bz therapy restored the expression of genes related to inflammatory response, cellular development, growth, and proliferation, and tissue development pathways, most probably linked to the cardiac remodeling processes inherent to CCC, thus mitigating the Th1-driven response found in vehicle-treated infected mice. The combined Bz+PTX therapy revealed pathways related to the modulation of cell death and survival, and organismal survival, supporting that this strategy may mitigate the progression of CCC. Altogether, our results contribute to the better understanding of the molecular mechanisms of the immune response in the heart tissue in chronic Chagas disease and reinforce that parasite persistence and dysregulated immune response underpin CCC severity. Therefore, Bz and Bz+PTX chemotherapies emerge as tools to interfere in these pathways aiming to improve CCC prognosis.

## Introduction

Chagas disease (CD) or American trypanosomiasis is an infectious disease caused by the protozoan parasite *Trypanosoma cruzi*, responsible for an estimated 6 to 7 million people infected and approximately 75 million at risk of infection (World Health Organization, 2021). Chagas disease was originally endemic in rural areas of Latin American countries, where Argentina, Brazil, Mexico, and Bolivia remain with the highest prevalence of infected people (Alarcón de Noya and Jackson, 2020). However, as globalization and migration mobility accelerated over the years, CD has disseminated to countries not originally endemic, such as United States, Spain, Japan, and Australia (Rassi et al., 2012; Antinori et al., 2017; Nunes et al., 2018; Bern et al., 2019; Alarcón de Noya and Jackson, 2020). Health care of currently infected individuals entails an expense of $7 billion annually, which poses as an enormous economic burden on global public health (Lee et al., 2013; Alarcón de Noya and Jackson, 2020). Chagas disease has a 4- to 8-week acute phase, characterized by patent parasitemia and inflammation due to tissue parasitism (Nunes et al., 2018), usually asymptomatic or causing broad symptoms manifestations as fever, enlargement of lymph nodes, and subcutaneous edema, which jeopardize a clear diagnosis (Rassi et al., 2012). Trypanocidal pharmacotherapy at this point is effective with a cure rate of 60% to 85% (Bern, 2011); the main problem, however, lies in diagnosing the disease and treating it at the right time. Untreated patients progress to the chronic phase after 2 to 3 months of infection, when parasitemia is mostly undetectable and, subsequently, most patients (60–70%) develop the indeterminate form and may show no clinical signs of CD throughout their lifespan. On the other hand, it is suggested that up to 5% of patients evolve each year to a clinical determinate form of CD, with 30% to 40% of the patients progressing to the chronic determinate forms of CD, with cardiac, digestive, or cardio-digestive manifestations (Sabino et al., 2013; Nunes et al., 2018).

The cardiac form, also known as chronic Chagas cardiomyopathy (CCC) is the most frequent and severe form of CD, being one of the main causes of morbidity and mortality due to heart diseases in endemic areas (Rassi et al., 2012). CCC onset may rely on unbalanced *T. cruzi* parasite*-*host immune response and disruption of intrinsic heart factors (Nunes et al., 2018), as CCC establishment and severity are characterized by the presence of inflammatory process associated with parasite persistence, leading to tissue injury, remodeling, and fibrosis, causing progressive alterations on cardiac rhythm, ventricular dysfunction, aneurysms, and thromboembolic events (Cunha-Neto and Chevillard, 2014; Nunes et al., 2018). In CD, inflammatory processes have been associated with alterations of cardiac electrophysiological properties, leading to changes in the electrocardiogram (ECG) records, such as increased corrected QT interval (QTc) (Eickhoff et al., 2010), leading to a condition known as prolonged QTc interval syndrome (Salles et al., 2003; Lazzerini et al., 2015). The prolonged QTc and most of the abnormalities associated with this syndrome are detected in the murine CCC model we used in the present study (Pereira et al., 2014a; Pereira et al., 2014b), making it attractive to investigate new therapeutic approaches and elucidate the molecular mechanisms and signaling pathways that might underpin the CCC onset and severity progression.

The two drugs currently used for the etiological treatment of CD, benznidazole (Bz) and nifurtimox, are effective when administered in the acute phase of infection (Dias et al., 2016). However, Bz has considerably reduced efficacy in the chronic phase, and controversial data support or do not support the efficacy of Bz in increasing negative seroconversion therefore interrupting the progression of ongoing CCC (Viotti et al., 2006; Bern, 2011; Machado-de-Assis et al., 2013; Morillo et al., 2015; Rassi et al., 2017). Benznidazole, a nitroimidazole derivative, has been used for CD treatment in the last 70 years, but relevant studies concerning its pharmacokinetics and biodistribution in humans and mice emerged only in the last decade (Soy et al., 2015; Perin et al., 2017; Perin et al., 2020), showing how we still lack information regarding its mechanism of action and triggered pathways. It is known that Bz is a prodrug, which exerts its effect after activation by the type I trypanosomal nitro-reductase enzyme, inherent to *T. cruzi* and other protozoa, thus producing reactive metabolites that have trypanocidal effect in the intracellular and extracellular forms of the parasite (Bern, 2011; Kratz et al., 2018). In addition, Bz is usually associated to a high rate of adverse events and lack of compliance with the 60-day standard-of-care (SoC) treatment, leading to permanent interruption of treatment in 20% to 30% of patients (Bern, 2011; Urbina, 2015). The BENEFIT study, the most extensive clinical trial with patients with CCC performed to date, showed that 192 (13.4%) of patients interrupted treatment during the study due to drug adverse events (AEs) (Morillo et al., 2015). Thus, in recent years, new clinical studies are making efforts to find new strategies to decrease Bz dosage and even increase its efficacy (Almeida et al., 2019; Molina-Morant et al., 2020a; Torrico et al., 2021) to reduce the occurrence of adverse events, thus enhancing treatment compliance (Ciapponi et al., 2020). On the other hand, efficacy of Bz treatment in progression of severity of CCC is still on debate (Rassi et al., 2017). Thus, further understanding of Bz molecular mechanisms and signaling pathways, especially in lower doses or dosing frequencies, is a matter of urgency in the field of CD translational research aiming to attain novel, safer, and more efficacious therapeutic strategies. Pentoxifylline (PTX) is a methylxanthine derivative with phosphodiesterase inhibitor activity, used on the treatment of peripheral vascular diseases, such as claudication, improving rheological properties of the blood (McCarty et al., 2016). Additionally, PTX has shown immunomodulatory and cardioprotective activities (Shaw et al., 2009). Treatment regimens with PTX, associated with traditional leishmanicidal drugs, have been studied on other parasites, such as *Leishmania* spp., mainly because development of cutaneous and mucosal leishmaniasis has been associated with immune dysregulation, with increased IFN-γ and TNF production (Brito et al., 2017; Faria et al., 2019). PTX has been explored by our group in the treatment of experimental CCC, and previously, we have shown that PTX reversed ECG abnormalities, reduced myocarditis and remodeling process, preventing progression of heart tissue injury and systemic immunological abnormalities (Pereira et al., 2015b). Moreover, we have proposed the Bz+PTX combined therapy, showing that it was able to reduce parasitemia, reverse ECG alterations and expression of tumor necrosis factor (TNF) in heart tissue, and its type-1 receptor (TNFR1) on T cells, as well as inducible nitric oxide synthase (iNOS/NOS2) in cardiac tissue, and NO concentrations in serum. Most importantly, the combination Bz+PTX sustained the beneficial effects in controlling parasitic load and reducing electrical abnormalities even after therapy discontinuation (Vilar-Pereira et al., 2016). However, the molecular mechanisms supporting these beneficial effects remain to be unveiled.

Previous investigations in gene expression profiling of transplanted human hearts with CCC identified immune response as one of the most upregulated pathways consistent with myocarditis and showed that IFN-γ was responsible in inducing several of the upregulated genes found dysregulated on CCC (Cunha-Neto et al., 2005). Conversely, expression profile in murine model of CCC revealed upregulation on genes mainly related to transcription factors, heart metabolism, energetics, and genes related to immune response included mainly secretory leukocyte protease inhibitor (SLPI), growth factor genes, extracellular matrix components, as well as adhesion molecule ICAM2 and CD24 (Mukherjee et al., 2003). Another study also investigating the expression profile of genes in the cardiac tissue of chronically *T. cruzi*–infected C57BL/6 mice showed that genes related to immune/inflammatory response and chemokine/cytokine receptor activity were the most activated pathways (Soares et al., 2010). Lastly, a recent phosphoproteomic study of heart tissue of experimental CCC showed that one of the most activated pathways was related to secreted immune effectors (Wozniak et al., 2020). Altogether, these findings support that genes associated with the immune response may play a pivotal role in the onset and progression of CCC.

Since molecular mechanisms in the immune response play an important part in CD, in the present study, using a well-described experimental model reproducing pivotal aspects of the cardiac form of CD in C57BL/6 mice infected with the *T. cruzi* Colombian (TcI) strain (Zingales, 2018), we investigate the immune molecular mechanisms involved in the CCC, using a RT-qPCR TaqMan array to evaluate the expression of 92 genes related to the immune response. Moreover, the immune mechanisms triggered by *T. cruzi* infection and reversed by treatment using a suboptimal dose Bz and the Bz+PTX combined therapy were explored.

## Materials and Methods

### Ethical Statements

This study was carried out in strict accordance with recommendations in the Guide for the Care and Use of Laboratory Animals of the Brazilian National Council of Animal Experimentation (https://www.mctic.gov.br/mctic/opencms/institucional/concea) and the Federal Law 11.794 (8 October 2008). The Institutional Committee for Animal Ethics of Fiocruz (CEUA-Fiocruz L004/09 and LW10/14) approved all experimental procedures used in the present study. All presented data were obtained from two independent experiments (Experiment Register Book #49, #53, and #57, LBI/IOC-Fiocruz).

### Experimental *T. cruzi* Infection and Drug Treatment

Mice obtained from the animal facilities of the Oswaldo Cruz Foundation (ICTB/Fiocruz, Rio de Janeiro, Brazil). Immediately after arrival, mice were housed in polypropylene cages under specific pathogen-free, randomly grouped in three mice per cage. The cages were maintained in microisolators, in standard conditions (with temperature and relative humidity of ~22°C ± 2°C and 55% ± 10%, respectively), noise and light (12-h light-dark cycle) control, and mice received grain-based chow food and water *ad libitum*. To minimize stress, mice were kept in adaptation for 10 to 14 days in a plastic igloo-enriched cage. After adaptation, 5- to 7-week-old female C57BL/6 (H-2^b^) mice were intraperitoneally infected with 100 blood-derived trypomastigotes (BDTs) of *T. cruzi* Colombian strain (DTU TcI) in 0.2 ml of vaccine-grade sterile buffered saline (BioManguinhos/Fiocruz, Brazil). After 120 days postinfection (120 dpi), all animals were in the chronic phase and presenting clinical signs of CCC (Silverio et al., 2012; Pereira et al., 2014a; Vilar-Pereira et al., 2016), receiving intraperitoneal injection with apyrogenic saline (non-infected, vehicle-treated group) or saline-containing PTX (Trental, Sanofi-Aventis) at 20 mg/kg and/or one quarter of Bz therapeutic dose (¼ Bz; 25 mg/Kg/day; LAFEPE) by gavage using apyrogenic water (BioManguinhos/Fiocruz, Brazil), daily, for 30 days. Cardiac alterations were monitored by electrocardiogram (ECG), before (120 dpi) and after (150 dpi) therapy. After euthanasia, cardiac tissues were collected in RNA later stabilization solution (Invitrogen, USA) for processing before molecular assays.

### ECG Registers

Mice were tranquilized with diazepam (10 mg/kg), and transducers were placed subcutaneously (DII). The traces were recorded for 2 min using a digital Power Lab2/20system connected to a bio-amplifier at 2 mV for 1 second (PanLab Instruments, Spain). The filters were standardized between 0.1 and 100 Hz, and the traces were analyzed using Scope software for Windows V3.6.10 (PanLab Instruments, Spain). The ECG parameters were analyzed as previously described (Silverio et al., 2012).

### Immunohistochemistry

At 150 dpi, mice were euthanized under anesthesia (ketamin 300 mg/kg + xilazin 30 mg/kg), and the hearts were removed, embedded in tissue-freezing medium (Tissue-Tek, Miles Laboratories, USA), and stored in liquid nitrogen. Serial cryostat sections, 3-μm thick, were fixed in cold acetone and subjected to polyclonal rabbit antibody recognizing mouse fibronectin (FN) (Gibco-BRL, USA) used at the dilution of 1:800 followed by indirect immunoperoxidase anti-rabbit immunoglobulin and peroxidase-streptavidin complex (Amersham, UK) staining revealed with liquid DAB+substrate chromogen system (DAKO, USA). Appropriate controls were prepared by replacing primary antibodies with immunoglobulin or normal rabbit serum. All antibodies and reagents were utilized in compliance with the manufacturer’s instructions. Images were digitized using a color view XS digital video camera adapted to a Zeiss microscope and analyzed with the Nis-Elements BR 4.0 software (Nikon Corporation, Tokyo, Japan). The FN-positive areas in 25 fields (12.5 mm^2^) per section (three sections per heart) were evaluated as previously described (Pereira et al., 2015a).

### DNA Extraction and *T. cruzi* Parasite Load Quantification by Quantitative Real-Time PCR

Genomic DNA was extracted from 10 to 20 mg of mouse hearts using High Pure PCR Template Preparation Kit (Roche Diagnostics, Indianapolis, IN), following the manufacturer’s instructions. Before extraction, tissues were withdrawn from RNA later and disrupted in 500 µl of tissue lysis buffer, using a TissueRuptor II (QIAGEN, USA) on maximal speed for 30 s. This homogenate was submitted to DNA extraction, according to the manufacturer’s recommendations. At the last step of the protocol, DNA was eluted from the silica column in 100 µl of elution buffer and stored at −20°C until further analysis. Amplification of *T. cruzi* satellite DNA was done by using the specific primers (Piron et al., 2007; Duffy et al., 2013) Cruzi1 (5′-ASTCGGCTGATCGTTTTCGA-3′) and Cruzi2 (5′-AATTCCTCCAAGCAGCGGATA-3′), both at 750 nM, and the TaqMan probe Cruzi3 (6FAM–CACACACTGGACACCAA–NFQ–MGB) at 50 nM. As an endogenous internal control, the predesigned TaqMan assay targeting mouse GAPDH gene (Cat no Mm99999915-g1, Applied Biosystems) was used. Standard curves were done spiking 1 × 10^6^ BDTs (Colombian strain), obtained from VERO cells infection, in 30 mg of non-infected heart tissue, proceeding DNA extraction, and making a 1:10 serial dilution of the eluted DNA in TE buffer, ranging from 10^6^ to 0.1 parasite equivalents. Real-time PCR reactions were carried out on Applied Biosystems ViiA 7 real-time PCR (Thermo Fisher, USA) thermocycler, using the cycling conditions: 50°C for 2 min, 94°C for 10 min, followed by 40 cycles at 95°C, and 58°C for 1 min, where fluorescence was collected after each cycle. All samples were run in duplicate, and threshold was set at 0.02 for both targets.

### Total RNA Extraction

Mouse hearts were withdrawn from RNA later and disrupted in 500 µl of lysis buffer using TissueRuptor II (QIAGEN, USA) on maximal speed for 30 s. Total RNA was extracted using mirVana™ miRNA Isolation Kit (Life Technologies), according to the manufacturer’s recommendations. Total RNA quantification and purity were assessed in a NanoDrop^®^ ND2000 (ThermoFisher), and integrity was analyzed in a Bioanalyzer 2100 (Agilent, USA) using RNA Nano 6000 kit. Only samples with RIN ≥ 7.5 were used in this study.

### Immune Response mRNAs Expression Profiling by Quantitative Real-Time PCR

A pool of three total RNA, extracted from cardiac tissue of infected and treated mice per group (non-infected, vehicle-treated, and Bz and Bz+PTX), were used for the gene expression analysis of 92 immune response and 4 endogenous genes according to the Applied Biosystems protocols. Reverse transcription was performed from 2.5 µg of total RNA using SuperScript™ IV VILO™ Master Mix with ezDNase (Invitrogen, USA), according to manufacturer’s instructions. Quantitative real-time RT-qPCR was done in 96-well pre-made TaqMan™ Array Mouse Immune Response (Applied Biosystems, ThermoFisher Scientific, USA/Cat no. 4414079). Each plate contained FAM/NFQ-MGB labeled TaqMan probes specific to 92 immune response genes and 4 endogenous reference gene candidates for data normalization and relative quantification. Ten microliters of Mastermix and 2 µl sample were loaded on each well, sealed, and centrifuged at 500*g* for 2 min. Real-time PCR reactions were carried out on Applied Biosystems ViiA 7 Real-Time PCR (Thermo Fisher, USA) thermocycler, using the cycling conditions: 10 min at 95°C, followed by 40 cycles of 15 s at 95°C and 60 s at 60°C. Fluorescence was collected after each cycle, at the annealing/extension step. Raw data files were pre-processed using QuantStudio™ Real-Time PCR Software (Applied Biosystems, USA) with threshold and baseline corrections for each sample, and gene expression results were analyzed using Expression Suite v1.0.3 (Applied Biosystems, USA). Threshold was set at 0.01 for all targets. After the stability score analysis of the reference gene candidates, using the Expression Suite Software, GAPDH and HPRT targets were selected as the most-stable reference gene pair. Gene expression was estimated by the ΔΔCt method (Livak and Schmittgen, 2001; Schmittgen and Livak, 2008), using uninfected samples as calibrators.

### Network Pathway Analysis

QIAGEN’s Ingenuity^®^ Pathway Analysis (IPA) software (build version 389077M, released 2019-08-30, content version 27821452, Qiagen, Redwood City, CA) (Inada et al., 2008; Helleman et al., 2010; Ngwa et al., 2011) was used for pathway analysis. IPA is a web-based application for data analysis in pathway context. Lists of differentially expressed genes and their respective fold change values were uploaded and used as input for the QIAGEN IPA. Genes were mapped to the IPA knowledgebase using the gene ids, and core analysis was performed to identify canonical pathways and top diseases and functions. “Ingenuity Knowledge Base (Genes + Endogenous chemicals)” was used as the reference set for “Expression analysis.” The analysis was based on “Expr fold change.” The default maximum and minimum values selected by IPA were used. Expression analyses were run with all analysis-ready molecules to identify important canonical pathways. Once a pathway is selected, IPA offers several options to view different items within the top significant pathways, such as horizontal and vertical bar charts. Top five gene networks for “Diseases and Functions” based on network score and focus molecules from the “Expression analyses” were identified. Bar graphs for activated and inhibited canonical pathways, diagrams for relevant pathways, and lists of molecules associated with the canonical pathways and diseases and functions were downloaded directly from the IPA network visualization tool.

### Analysis of Individual Gene Expression by RT-qPCR

For gene expression assessment of individual mRNA targets, predesigned TaqMan^®^ Assays were used, according to the manufacturer’s recommendations: IFN-γ (assay ID Mm00801778_m1), CD3e (assay ID Mm00599683_m1), IL-2 (assay ID Mm00434256_m1), CSF2 (assay ID Mm00438328_m1), IL-7 (assay ID Mm00434291_m1), C3 (assay ID Mm00437858_m1), CD19 (assay ID Mm00515420_m1), and Selectin (assay ID Mm00441278_m1). As reference genes, GAPDH (assay ID Mm99999915_g1) and HPRT (assay ID Mm00446968_m1) were used. Two µg of total RNA was treated with DNAse I (Sigma) following manufacturer’s recommendations, and reverse transcription was subsequently performed using Superscript IV (Invitrogen) with random primers. Real-time quantitative RT-PCR was carried out in a 10-µl reaction containing 5 µl 2× TaqMan Universal PCR Master Mix (Applied Biosystems), 0.5 µl 20× TaqMan probe, 2 µl cDNA (at the concentration of 1 ng/µl), and 2.5 µl of nuclease-free water. Real-time PCR reactions were carried out on Applied Biosystems ViiA 7 Real-Time PCR System (Thermo Fisher, USA) using the cycling conditions: 10 min at 95 °C, followed by 40 cycles of 15 s at 95°C and 60 s at 60°C. Fluorescence was collected after each cycle at the annealing/extension step. All samples were run in duplicate, and threshold was set at 0.02 for all targets. Results were analyzed using ExpressionSuite v1.0.3 (Applied Biosystems, USA), GAPDH and HPRT targets were selected as endogenous controls, and gene expression was estimated using the ΔΔCt method (Livak and Schmittgen, 2001; Schmittgen and Livak, 2008). Relative quantification was estimated using NI animals as calibrators, except for the cytokine IL-2, because there was no amplification for the NI group, an animal of NI+Bz group was used as a calibrator.

### Statistical Analysis

Apart from the RT-qPCR TaqMan array, which was run only once using blood pooled from three mice, all experiments were performed at least in three technical replicates. The threshold cycle method was used to calculate the relative mRNA expression after global normalization. The hierarchical clustering was performed using squared Euclidean as distance measure and Ward’s method for linkage analysis and z-score normalization. For gene expression analysis by RT-qPCR, normality test was done by Shapiro-Wilk test followed by one-way ANOVA (two-tailed hypothesis test) all pairwise multiple comparison (Tukey, Bonferroni or Dunn’s test) with SigmaPlot for Windows version 12.0 (Systat Software, Inc). Results were expressed as means and standard deviations (SDs), differences were considered significant if p < 0.05 as described in each figure legend.

## Results

### Chronic Model of CD Following Bz and/or PTX Treatment

**Figure 1:**
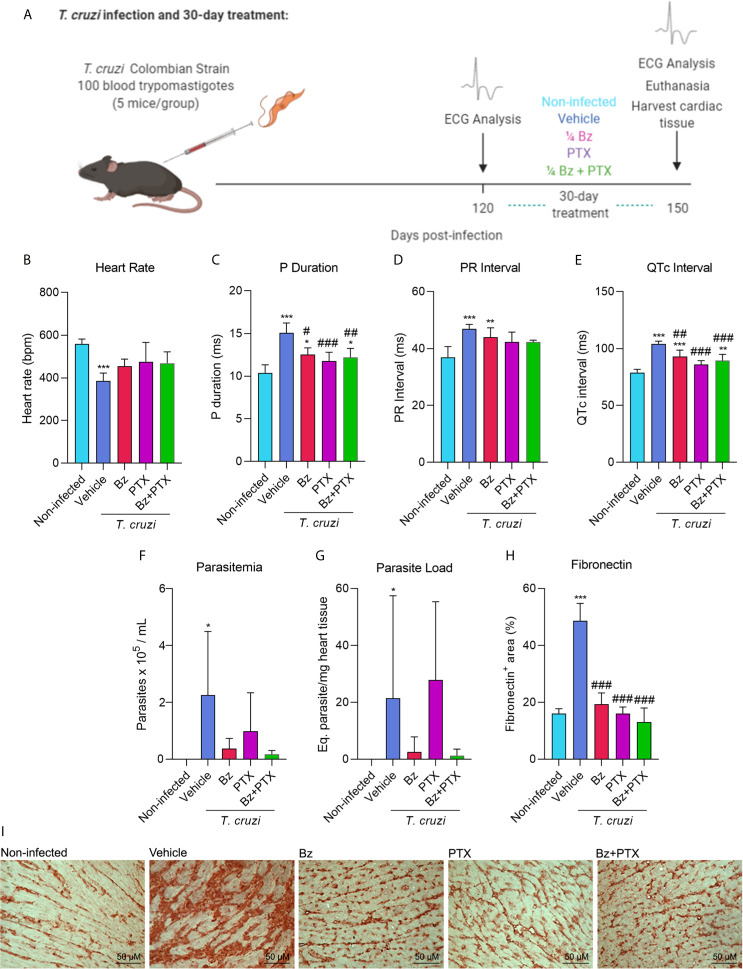
Chronic Chagas cardiomyopathy model and treatment with suboptimal dose of benznidazole (Bz), pentoxifylline (PTX), or benznidazole and pentoxifylline (Bz + PTX). **(A)** Experimental design of infection and treatment. Mice were infected and treated daily from 120 to 150 dpi with vehicle, suboptimal dose of Bz (¼ dose, 25 mg/kg), PTX (20 mg/kg), a combined therapy of Bz+PTX and analyzed at 150 dpi. **(B)** Average heart rate (*vs*. non-infected: vehicle: p = 0.001; Bz: p = 0.047). **(C)** Average P duration (*vs*. non-infected: vehicle: p < 0.001; Bz: p = 0.021; *vs*. vehicle: Bz: p = 0.010; PTX: p < 0.001; Bz+PTX: p = 0.003). **(D)** Average PR interval (*vs*. non-infected: Vehicle: p < 0.001; Bz: p = 0.008). **(E)** Average QTc interval (*vs*. Non-infected: Vehicle: p < 0.001; Bz: p < 0.001; Bz+PTX p = 0.006; *vs*. vehicle: Bz: p = 0.008; PTX: p < 0.001; Bz+PTX: p < 0.001). **(F)** Parasitemia levels (*vs*. non-infected: vehicle: p < 0.05). **(G)** Parasite load based on qPCR detection of T. cruzi satellite DNA on mice heart tissue (*vs*. Non-infected: Vehicle: p < 0.05). **(H)** Percentage of fibronectin+ area (*vs*. non-infected: vehicle: p < 0.001; *vs*. vehicle: Bz: p < 0.001; Bz+PTX p < 0.001; Bz+PTX: p < 0.001). **(I)** Immunohistochemical staining of fibronectin in the cardiac tissue. Number of mice per group: non-infected = 5; Vehicle = 4; Bz = 5; PTX = 5; Bz+PTX = 5. For all graphs, significance was determined using one-way ANOVA all pairwise multiple comparison *vs*. non-infected (*p < 0.05, **p < 0.01, ***p < 0.001) and *vs*. Vehicle (^#^p < 0.05, ^##^p < 0.01, ^###^p < 0.001).

To determine the effect of suboptimal dose of Bz and/or PTX on the molecular pattern of immune response in experimental CD heart disease, C57BL/6 mice were intraperitoneally infected with 100 BDTs (Colombian strain), which is a highly pathogenic parasite strain. This mouse model of CCC was first established by Silverio et al. (2012) and successfully reproduced in several studies (Pereira et al., 2014a; Pereira et al., 2014b; Pereira et al., 2015a; Pereira et al., 2015b; Vilar-Pereira et al., 2016; Vilar-Pereira et al., 2021). After 120 dpi, when electrical abnormalities and heart injury were already installed, mice were treated with vehicle (saline injection/water gavage), Bz (25 mg/kg/day), PTX (20 mg/kg/day), or the combined dose of Bz and PTX (Bz+PTX) for 30 consecutive days (**Figure 1A**). At 150 dpi, relevant electrical abnormalities could be observed in vehicle-treated animal group that presented bradycardia (**Figure 1B**), arrythmias shown in altered P duration and PR interval (**Figures 1C, D** **)**, and a prolonged QTc interval when compared with the non-infected (NI) mice group (**Figure 1E**). Parasites could also be detected by RT-qPCR in the peripheral blood of vehicle-treated group (**Figure 1F**) and in the heart tissue (**Figure 1G**). After 30 days of drug therapy, treatments with Bz, PTX, or Bz+PTX partially reversed electrical abnormalities found in the vehicle-treated group and notably, no parameter was aggravated by the three therapeutic schemes used (**Figures 1B–E**). Bz treatment alone was effective, partially reversing arrythmias as shown in P duration (**Figure 1C**) and QTc interval (**Figure 1E**) and controlling *T. cruzi* growth even in a suboptimal dose, as shown by the reduced parasite load in the blood (**Figure 1F**) and heart tissue (**Figure 1G**). However, this same outcome could not be seen for PTX therapy alone, which failed in controlling parasite load in peripheral blood (**Figure 1F**) and heart tissue (**Figure 1G**). Crucially, the combined therapy with Bz+PTX was also effective in controlling the parasite load in both blood and heart tissue (**Figures 1F, G** **)**, indicating that associated PTX did not abrogate the trypanocidal effect of Bz and could potentially be used in a combined chemotherapy strategy to hamper CCC progression. The deposition of the extracellular matrix component FN in the cardiac tissue reveals fibrosis, a feature of chronic Chagas heart disease (Rassi et al., 2012). At 150 dpi, compared with non-infected controls, chronically infected C57BL/6 mice showed increased deposition of FN in the heart tissue and crucially, the overdeposition of FN reduced after therapies with Bz, PTX, and Bz+PTX (**Figures 1H, I** **)**.

### Overview of Immune Array Results

**Figure 2:**
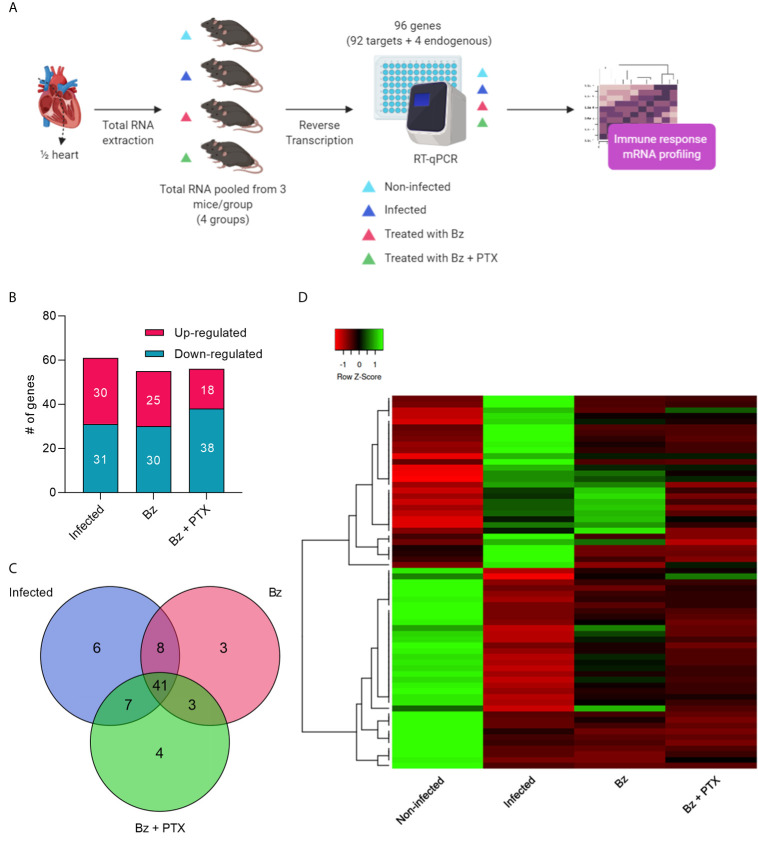
Overview and characterization of immune array analysis. **(A)** Schematic design of array workflow. Mice cardiac tissues were harvested 30 days after therapy and ½ of the heart was submitted to total RNA extraction, subsequent reverse transcription, and RT-qPCR of 92 immune-related genes (92 targets + 4 endogenous controls). **(B)** Number of altered genes in each group. **(C)** Venn diagram showing the number of altered expressed genes in each group. **(D)** Heatmap and hierarchical clustering. The color scale illustrates gene’s fold change after global normalization; red represents −1 to 0 and green is 0 to 1. Clustering was performed using average linkage distance measurement method: Spearman Rank correlation. #: number.

Our applied RT-qPCR TaqMan array workflow (**Figure 2A**) assessed 92 immune response genes in RNA of mouse hearts pooled from three sorted mice/group by RT-qPCR. Of 92 genes, 80 showed amplification on every group, and 12 showed no amplification in any of the groups analyzed. Genes altered by at least two-fold change were selected, revealing an intensely dysregulated immune response for the vehicle-treated group, which showed 61 altered genes (30 up and 31 downregulated; **Table S1**) and 19 unaltered genes (**Figures 2B** and **S1A**). Therapy with Bz showed 55 (25 up and 30 downregulated) altered and 25 unaltered immune genes (**Figures 2B** and **S1B**), whereas Bz+PTX revealed 56 (18 up and 38 downregulated) altered genes with 24 unaltered ones (**Figures 2B** and **S1C**). Overall, we found altered expression for 71 immune genes in either *T. cruzi* infection or drug therapies groups, and they showed high overlap, with 41 altered genes being shared in between groups (**Figure 2C**). Hierarchical clustering revealed that the gene expression profiles for each one of the groups clustered independently (**Figure 2D**), specially the non-infected and vehicle-treated groups.

### Chronic CD Cardiomyopathy Induces a Dysregulated and Persistent Immune Response

**Figure 3:**
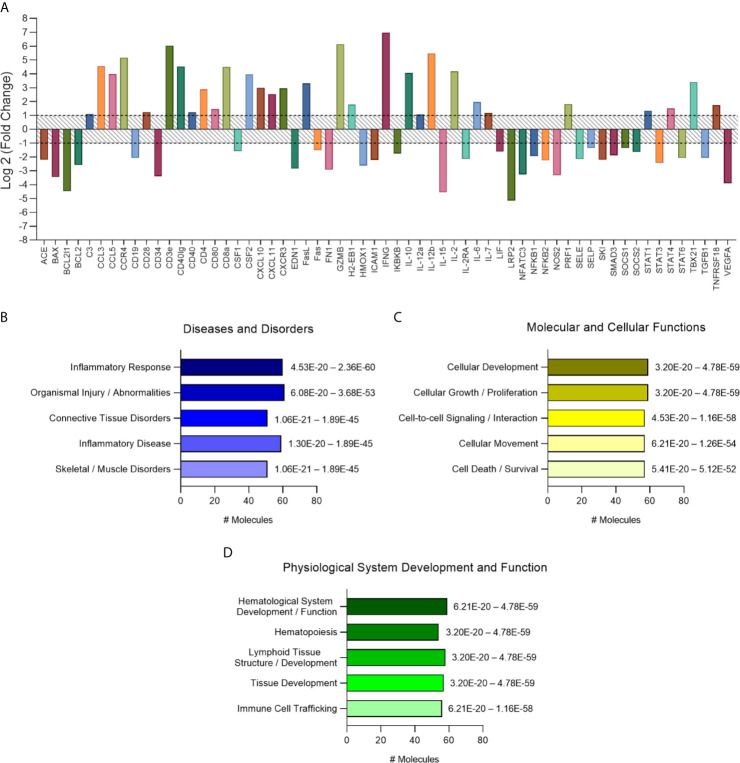
Characterization of immune response expression profile for the chronically infected group. **(A)** Overall expression of the altered immune response genes found in this study. Results were expressed as Log2 (Fold Change) and genes with fold change values under -1 (fold change 0.5) and over 1 (fold change 2) were considered altered and used for further analysis on IPA software. **(B)** Diseases and disorders pathway analysis. **(C)** Molecular and Cellular Function pathway analysis. **(D)** Physiological System Development and Function pathway analysis. Significance interval of enrichment is shown on the right of the bars. #: number.

The analysis of altered genes in the vehicle-treated group of mice was performed in comparison to the non-infected controls. Genes presenting two-fold change (up- or downregulated) from *T. cruzi*–infected group (**Figure 3A**) were analyzed using Ingenuity Pathway Analysis Software (QIAGEN, USA). Meaningful interpretation of gene-expression data is facilitated by prior biological knowledge. Expression changes observed in data sets can be explained by causal networks constructed from individual relationships curated from prior knowledge (Krämer et al., 2014). The application of causal networks integrating previously observed cause–effect relationships reported in the literature have become common (Pollard et al., 2005; Kumar et al., 2010; Chindelevitch et al., 2012; Martin et al., 2012; Felciano et al., 2013). Thus, molecular pathway analysis revealed that regarding diseases and disorders pathways, most genes were associated with “inflammatory response,” followed by “organismal injury and abnormalities” (**Figure 3B**), which appears to be driven mainly by the T-cell growth promoter cytokine IL-2 (18.2-fold change) and the pro-inflammatory cytokines IL-12b and IFN-γ (44.3- and 126.5-fold change, respectively), the most altered cytokines in the heart tissue of vehicle-treated group (**Table S1**). The T-cell CC chemoattractants CCL3 and CCL5 (23.4- and 15.7-fold change, respectively) may also contribute to the “inflammatory response” activated pathway (**Figure 3B**). As to molecular and cellular functions, pathways related to “cellular development,” “cellular growth and proliferation,” “cell-to-cell signaling and interaction,” “cellular movement and cell death and survival” are the most activated pathways (**Figure 3C**). Among physiological system development and function related pathways “hematological system development and function” and “tissue development and immune cell trafficking” were the most important pathways to be activated (**Figure 3D**). These were possibly driven by the increased expression of CSF2 (granulocyte-macrophage colony-stimulating factor), controlling the production, differentiation, and function of granulocytes and macrophages, and thereby increasing activation of hematopoiesis. Analysis of the pathways related to the top diseases and functions revealed an activation of “Th1/Th2 pathway,” followed by “type I diabetes mellitus signaling,” “altered T and B cell signaling in rheumatoid arthritis,” and “T helper cell differentiation” (**Table 1**).

**Table 1:** Top disease and functions gene networks on regulated immune genes for the chronically infected group identified by the IPA software.

Top Diseases and Functions	Score	Focus molecules	Molecules in Network
Cell-to-cell signaling and interaction, hematological system development and function, lymphoid tissue structure and development	24	12	Arginase, CD4, CD40, CD8, CD8A, Ctbp, Cyclooxygenase, CYP, IFN, IFN Beta, IFNG, IgE, IL-12 (family), IL12B, IL2RA, immune complex, Interferon alpha, LIF, MHC Class I complex, MHC CLASS I (family), MHC Class II (complex), Mst/krs, NAD, NFKB2, NGF, NOS, Pias, Pro-inflammatory Cytokine, SOCS1, SOCS2, STAT5 a/b, STAT6, TLR, TLR2/3/4/9, TNF (family)
Cell Death and Survival, Free Radical Scavenging, Molecular Transport	19	10	26s Proteasome, ADRB, Alp, Ant, BAX, BCL2, BCL2L1, Calcineurin A, Calcineurin protein(s), calpain, Caspase, CD40LG, CG, creatine kinase, CSF2, CXCL10, Cytochrome C, Cytochrome-c oxidase, DFF, FAS, FasLg, Growth factor, Hdac, HISTONE, Hsp70, Hsp90, IL1, ITPR, LDL-cholesterol, NOS2, Notch, P38 MAPK, Rb, Vdac, VEGFA
Cardiovascular disease, organismal injury and abnormalities, renal and urological disease	12	7	ACE, Alpha Actinin, Alpha catenin, Alpha tubulin, CD34, Collagen, Collagen type I (complex), Collagen type II, Collagen type III, Collagen type IV, Collagen(s), collagenase, ECM, elastase, FGF, Fibrin, Granzyme, GZMB, IL7, Integrin, Integrin alpha V beta 3, Kallikrein, Laminin (complex), MMP, PKG, PROTEASE, SELP, Serine Protease, SKI, SMAD, TGF-B, TGF-B1, TH1 Cytokine, Thrombospondin, Trypsin
Connective tissue disorders, inflammatory disease, inflammatory response	12	7	Ap1, Beta Arrestin, CCL5, CCR4, Chemokine, Chemokine receptor, Cyclin B, Cytokine, Focal adhesion kinase, G protein, GPCR, IKBKB, IL10, IL2, MAPK, MTORC1, NFKB1, P110, p85 (pik3r), Pdgfr, PI3Kγ, PTK, Rac, RAS, Ras homolog, Receptor protein tyrosine kinase, RNA polymerase II, Secretase gamma, SELE, Sfk, Shc, SRC (family), STAT, TCR, VEGF
Cell signaling, cell-to-cell signaling and interaction, hematological system development and function	10	6	14-3-3, Alpha actin, C-C chemokine receptor, Calcineurin B, Caspase 3/7, CCL3, CD28, CD3, CD3-TCR, CD3E, CXCL11, CXCR3, Gm-Csf Receptor, H/K/NRAS, HLA-DR, IFNG, IL-2R, IL-10R, IL-8r, Importin beta, JNK, Lamin b, Lfa-1, NADPH oxidase, NFAT (family), NFATC3, Pdgf (complex), PI3K (family), PI3K p85, Plc beta, Raf, Rap1, SAPK, SOS, TSH

**Figure 4:**
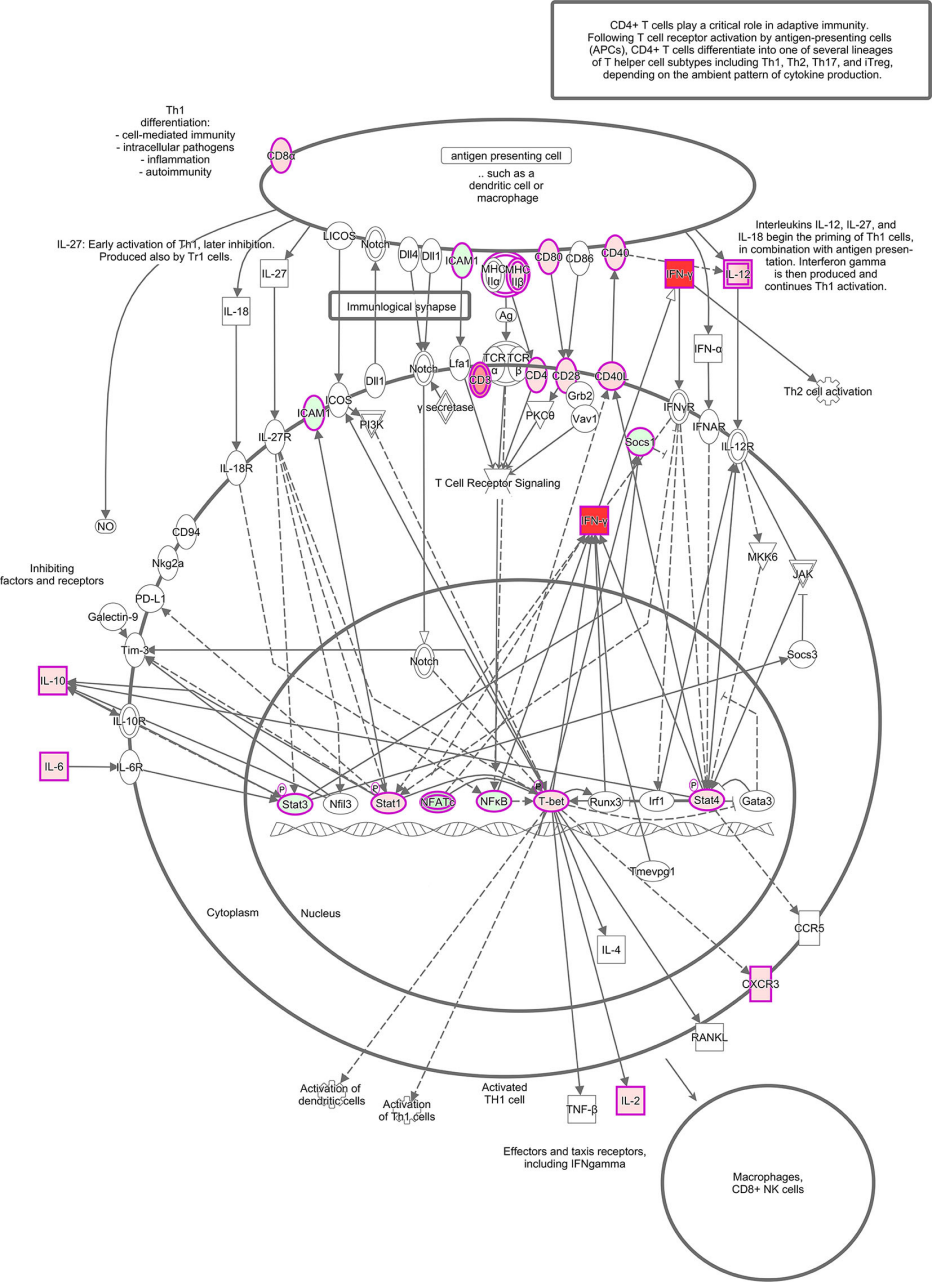
Th1 activation pathway representation. *In silico* analysis done using IPA software (QIAGEN, USA) showing a signaling pathway built with: IFN-γ, CD3, CD4, CD8α, CD28, CD40, CD40L, CD80, ICAM1, CXCR3, MHCIIβ, IL-12, IL-10, IL-6, IL-2, Socs1, Stat1, Stat3, Stat4, NFATϵ, NFκB, and T-bet. The molecules are highlighted with fuschia/pink outlines and filled with different shades of red or green color which indicate up and down-regulation, respectively, based on their fold change values from the infected group compared to the non-infected group. Direct line: direct activation; dashed line: indirect activation.

The canonical pathways revealed a long list of significant activated pathways for the vehicle-treated group based of the expression profile of immune genes. Among the most upregulated pathways (orange-shade bars), we can highlight “Th1 pathway,” “type I diabetes mellitus signaling,” “crosstalk between dendritic and natural killer cells,” “dendritic cell maturation,” and at a lower degree, “natural killer cells signaling,” “apoptosis signaling,” and “cytotoxic T lymphocyte apoptosis of target T-cells and interferon signaling” (**Figure S2**). This analysis also revealed downregulated pathways (blue-shade bars) that can be represented by “T-cell exhaustion signaling pathway,” “role of pattern recognition receptors of bacteria and viruses,” and at a lower degree “IL-15 signaling” (**Figure S2**). To represent one of the several upregulated pathways of this study, we are highlighting the Th1 pathway, which is highly activated during *T. cruzi* chronic infection (**Figure 4**), based on a previous study (Chevillard et al., 2018). In the schematic figure, we can see that antigen presentation by dendritic cells or macrophages, by priming Th1 cells *via* CD40/CD40L, CD80/CD28, and MHC-IIβ/CD4 (all with increased expression in this study) induce the production of pro-inflammatory cytokine IL-12 contributing to an amplification of Th1 activation by inducing the production of IFN-γ. IFN-γ production is also induced by transcription factors T-bet, Stat1, and Stat4 in the nucleus. Notably, increased expression IFN-γ by distinct molecular mechanisms can be associated with upstream regulators found in this study.

Finally, regarding top diseases and functions, it was observed that most molecules from this study were associated with “cell-to-cell signaling and interaction, hematological system development and function, lymphoid tissue structure and development.” Ten molecules were related to “cell death and survival, free radical scavenging, molecular transport,” “Cardiovascular disease, organismal injury and abnormalities, renal and urological disease” with seven molecules, “connective tissue disorders, inflammatory disease, inflammatory response” also with seven molecules, and “cell signaling, cell-to-cell signaling and interaction, hematological system development and function” with six relevant molecules were the other top diseases and functions (**Table 1**).

### Bz and PTX Combined Therapy Restores Expression of Relevant Immune Genes and Regulate Cell Survival Pathways

**Figure 5:**
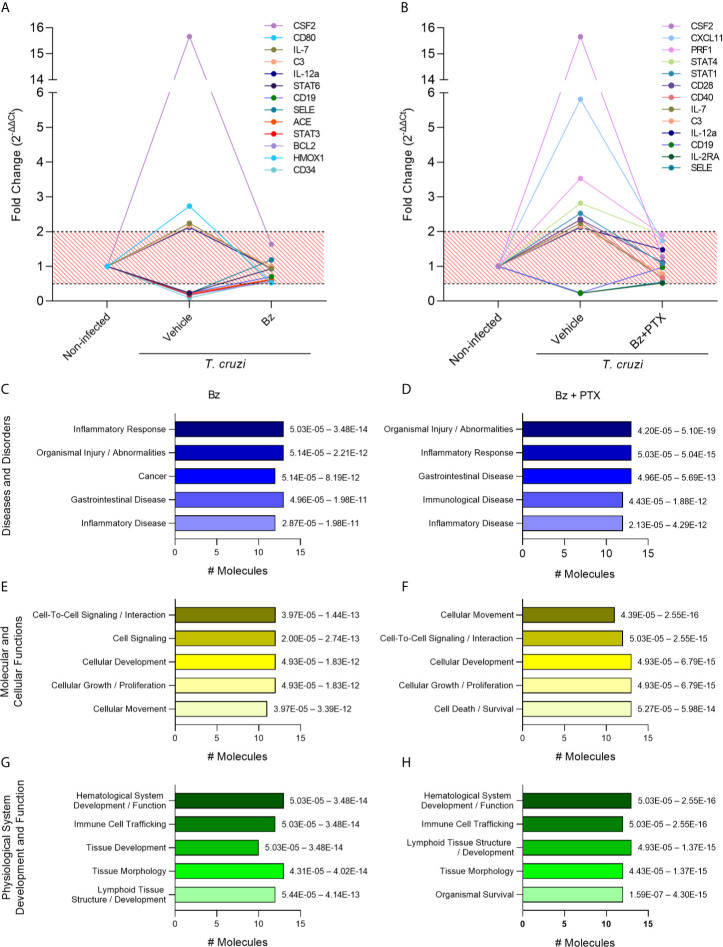
Characterization of immune response expression profile for the Bz and Bz + PTX group. **(A)** Expression of immune genes increased in the infected group and with restored expression with Bz therapy or **(B)** Bz+PTX therapy. Relative expression was expressed as fold change (2^-ΔΔCt^). Genes with values under 0.5 and over 2 in the infected group and in between 0.5 and 2 in Bz or Bz+PTX therapies group were considered as restored expression and selected for further analysis on IPA software. The graphs show the activated pathways in the infected group that are regulated with therapy. **(C, D)** IPA analysis with top significantly associated to Disease and Disorders to the Bz or Bz+PTX groups, respectively. **(E, F)** IPA analysis with top significantly associated to Molecular and Cellular Functions to the Bz or Bz+PTX groups, respectively. **(G, H)** IPA analysis with top significantly associated to Physiological System Development and Function to the Bz or Bz+PTX groups, respectively. The significance interval of enrichment is shown on the right of the bars.

Following the immune molecular characterization of vehicle-treated group, we focused on the assessment of genes controlled by Bz or Bz+PTX therapies aiming to dissect the most relevant signaling pathways affected by the proposed treatments. For that, we selected the genes that had their expression restored upon therapy, meaning, the genes that were altered during *T. cruzi* infection (fold change of 2.0 or above) but had their expression restored (fold change below 2.0) with therapy using Bz or Bz+PTX. We were able to identify 13 genes that fulfill this criterion in the Bz-treated group (**Figure 5A**) and 13 genes in the Bz+PTX combined therapy group (**Figure 5B**). When subjected to pathway analysis, the Bz therapy revealed that most altered genes were regulating pathways mainly related to “inflammatory response/disease” and “organismal injury and abnormalities” (**Figure 5C**). Regarding molecular and cellular functions, Bz was acting on genes involved mostly in “cell-signaling, cellular development, growth and proliferation” and “cellular movement pathways” (**Figure 5E**). Physiological system development and function analysis revealed regulation of “hematological system development/function,” “immune cell trafficking,” and “tissue development/morphology” (**Figure 5G**). Finally, among the top canonical pathways, “T helper cell differentiation,” “B cell development,” “Th1 and Th2 activation,” and “hematopoiesis from pluripotent stem cells” seems to be the most regulated pathways upon Bz therapy (**Figure S3A**). Conversely, the Bz+PTX combined therapy showed few different regulated pathways, as it also acts regulating “organismal injury and abnormalities” and “inflammatory response” pathways (**Figure 5D**), followed by “cellular movement,” “cell-to-cell signaling and interaction,” “cellular development,” and “cellular growth and proliferation” (**Figure 5F**). Additionally, among physiological system development and function pathways, in addition to the pathways that were already identified in the Bz therapy, the combined therapy also revealed the “organismal survival” pathway, corroborating the role of PTX in cell death signaling pathways (**Figure 5H**). Ultimately, in top canonical pathways, besides acting on “T helper cell differentiation” and “Th1/Th2 activation pathways,” the “crosstalk between dendritic cells and natural killer cells” and “T-cell exhaustion” appeared as new activated pathways, showing the significance of PTX therapy to reverse this phenomenon triggered by the long-lasting *T. cruzi* infection, mainly by antigenic long stimulation. In the schematic figure for this pathway (**Figure S4**), based on the immune genes regulated by Bz+PTX in the vehicle-treated group, it is suggested that the upregulation of IL-12, CD28, CD80, STAT 1/2, and STAT 4, reversed by the combined therapy, is promoting a decrease in T-cell exhaustion and stimulating a more effective T-cell response.

### IFN-γ, CD3ε, IL-2, CSF2, and C3 Are Regulated Upon Etiological Therapy

**Figure 6:**
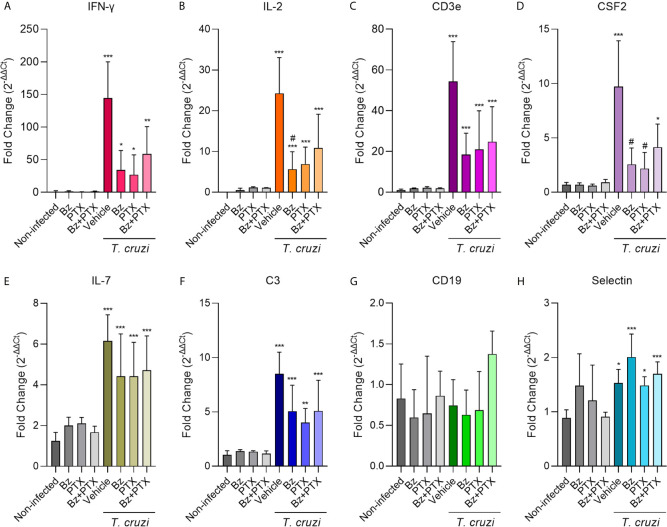
Validation of selected immune response genes on individual samples. **(A)** IFN-γ (vs. non-infected: vehicle: p < 0.001; Bz: p < 0.001; PTX: p < 0.001; Bz+PTX: p < 0.001), **(B)** IL-2 (*vs*. non-infected: vehicle: p < 0.001; Bz: p = 0.013; PTX: p = 0.047; Bz+PTX: p = 0.005; *vs*. vehicle: Bz: p = 0.030), **(C)** CD3ε (*vs*. non-infected: vehicle: p < 0.001; Bz: p < 0.001; PTX: p < 0.001; Bz+PTX: p < 0.001), **(D)** CSF2 (*vs*. non-infected: vehicle: p < 0.001; Bz+PTX: p = 0.028; *vs*. vehicle: Bz: p = 0.027; PTX: p = 0.042), **(E)** IL-7 (*vs*. non-infected: vehicle: p < 0.001; Bz: p < 0.001; PTX: p < 0.001; Bz+PTX: p = 0.001), **(F)** C3 (*vs*. non-infected: vehicle: p < 0.001; Bz: p < 0.001; PTX: p = 0.007; Bz+PTX: p < 0.001), **(G)** CD19 (*vs*. non-infected: ns; *vs*. vehicle: ns), and **(H)** selectin (*vs*. non-infected: vehicle: p = 0.013; Bz: p < 0.001; PTX: p = 0.034; Bz+PTX: p = 0.001) were assessed by real-time RT-qPCR in each group of the experiment. Number of mice per group: non-infected = 3/group; vehicle = 4; Bz = 5; PTX = 3; Bz+PTX = 5. The expression is shown as mean ± SD for each group by 2^-ΔΔCt^ relative quantification method. For all graphs, significance was determined using one-way ANOVA all pairwise multiple comparison *vs*. non-infected (*p < 0.05, **p < 0.01, ***p < 0.001; ns, non-significant) and *vs*. vehicle (#p < 0.05; ns, non-significant).

Aiming to validate the regulation of gene expression observed using the RT-qPCR TaqMan array, we selected eight genes to explore further on individual samples, based on the importance to *T. cruzi* infection establishment and on restored expression upon both therapies Bz and Bz+PTX on the TaqMan array (**Figure 6**). Based on available literature exploring the relationship of these genes with *T. cruzi* chronic infection, we first selected IFN-γ, IL-2, and CD3ε to validate and strengthen the reliability in our data. The expression of IFN-γ, a key inflammatory cytokine in CD (Cunha-Neto et al., 2009), was increased (144.40 ± 55.66) in the vehicle-treated group when compared with the non-infected mice (1.13 ± 1.31). Therapy with Bz was able to ease this alteration, decreasing at least three times the expression of IFN-γ (33.99 ± 30.02) and a trend to reduction of IFN-γ expression was also detected when mice were treated with PTX (26.95 ± 30.45) and Bz+PTX (58.84 ± 41.81) (**Figure 6A**). IL-2, a broad pro-inflammatory cytokine studied in CD (Briceño and Mosca, 1996; Abel et al., 2001; Mengel et al., 2016), showed a significant increase upon *T. cruzi* infection (24.27 ± 8.76) when compared to the non-infected group (not-detected), which was partially abrogated after Bz (5.66 ± 4.28), PTX (6.90 ± 4.16), and Bz+PTX therapies (10.88 ± 8.24) (**Figure 6B**). CD3ε, a subunit of T-cell receptor CD3 was also investigated and showed significant increase in the vehicle-treated group (54.35 ± 19.46) compared to the non-infected group (1.02 ± 0.45), which was partially abrogated upon Bz therapy (18.50 ± 10.42), PTX (20.92 ± 19.03), and Bz+PTX (24.74 ± 17.18) therapies (**Figure 6C**). For these three genes, the previous results using the RT-qPCR TaqMan array in the vehicle-treated group matched the validation results using the individual assays, corroborating the strength of our analysis.

Additionally, we selected five genes that were restored upon Bz and Bz+PTX therapies to validate our findings. CSF2 showed a significant increase (9.71 ± 4.21) compared with the non-infected group (0.68 ± 0.21). Interestingly, CSF2 expression was reversed through therapy with Bz (2.57 ± 1.51), PTX (2.18 ± 1.50) and partially reversed by Bz+PTX combined therapy (4.15 ± 2.12) (**Figure 6D**). IL-7 expression was also increased in chronically *T. cruzi*–infected mice (6.15 ± 1.29) when compared with the non-infected control (1.24 ± 0.42). In this situation, therapies with Bz (4.41 ± 2.08), PTX (4.42 ± 1.67), and Bz+PTX (4.72 ± 1.684) were unable to down-regulate the expression of this cytokine (**Figure 6E**). The complement protein C3 showed significantly increased expression in the vehicle-treated group (8.50 ± 2.00) compared with the non-infected control group (1.06 ± 0.37). Again, treatment with Bz (5.06 ± 2.40) and Bz+PTX (5.08 ± 2.83) was unable to down-modulate or restore C3 expression, although PTX (4.01 ± 1.30) therapy decreased C3 expression (**Figure 6F**). CD19, a B-lymphocyte antigen, had shown decreased expression in our RT-qPCR TaqMan array profiling for the vehicle-treated group. However, it was not confirmed by the validation of individual samples, as the vehicle-treated group (0.74 ± 0.32) showed no alterations in CD19 expression compared with non-infected control (0.83 ± 0.42). Moreover, Bz (0.63 ± 0.30), PTX (0.69 ± 0.47), and Bz+PTX (1.37 ± 0.28) therapeutic strategies showed no regulation of CD19 expression (**Figure 6G**). Selectin, a glycoprotein from the cell adhesion molecules family (Marchini et al., 2021), had shown decreased expression in our RT-qPCR TaqMan array profiling for the vehicle-treated group compared with the non-infected group. However, in the validation of individual samples, the expression of selectin increased in the vehicle-treated group (1.53 ± 0.25), when compared with the non-infected control (0.89 ± 0.15), and therapy with Bz (2.00 ± 0.42), PTX (1.48 ± 0.16) or the combined therapy with Bz+PTX (1.70 ± 0.22) have no impact in selectin expression in the heart tissue of chronically infected mice (**Figure 6H**).

## Discussion

Chronic Chagas cardiomyopathy is the most frequent and severe form of CD, causing high rates of morbidity in endemic and non-endemic areas (Pérez-Molina and Molina, 2018). There is a considerable amount of evidence supporting that dysregulation of the immune response in the chronic phase of infection improves risk of complications in CD (Perez-Fuentes, 2003; Guedes et al., 2016) is associated with the onset and severity of CCC (Cunha-Neto et al., 2005; Cunha-Neto et al., 2009; Cunha-Neto and Chevillard, 2014) and parallels with cardiac abnormalities related to a poor prognosis (Lazzerini et al., 2015; Chevillard et al., 2018). Although efforts have been made to understand the signaling pathways and molecular mechanisms underpinning CCC onset and progression to more severe stages (Mukherjee et al., 2003; Cunha-Neto et al., 2005; Soares et al., 2010; Ferreira et al., 2017), to date, there is no study showing the main immunological signaling pathways regulated by the trypanocidal drug Bz or other associated treatment. Our well-established experimental murine model reflects pivotal alterations found in human CCC (Brito and Ribeiro, 2018; Nunes et al., 2018), such as bradycardia, arrythmias, and prolonged PR and QTc intervals. Furthermore, the prolonged QTc interval, which reflects the duration of the conduction action potential in the ventricles (depolarization and repolarization), has been described as a mortality risk predictor in CCD patients (Salles et al., 2003). More recently, the prolonged QTc has been associated with a pro-inflammatory-enriched cardiac inflammatory process in several heart illnesses, supporting the proposal of a prolonged QTc syndrome (Lazzerini et al., 2015). Crucially, both prolonged PR and QTc intervals are reproduced in our experimental model of infection of C57BL/6 mice with the Colombian strain and, as predicted, associated with the presence of pro-inflammatory cytokines and chemokines in the heart tissue (Gibaldi et al., 2020).

Our data showed that suboptimal dose of Bz decreased parasitemia and heart parasite load, corroborating previous findings (Vilar-Pereira et al., 2016). Indeed, compared with the therapeutic dose (100 mg/kg/day), lower doses of Bz are able to control parasite burden *in vivo* and *in vitro* (Cevey et al., 2016), raising the question that the Bz dosing regimen currently used should be revised and optimized (Almeida et al., 2019; DNDi, 2019; Molina-Morant et al., 2020a; Torrico et al., 2021). Although Bz therapy has controversial data as a therapeutic approach to hamper progression of Chagas heart disease when used in chronically infected patients (Machado-de-Assis et al., 2013; Morillo et al., 2015; Rassi et al., 2017; Nunes et al., 2018), our data support that therapy with the suboptimal Bz dose, which was able to partially reverse pivotal electrical abnormalities (bradycardia, P duration, QTc). Therapy with PTX showed no effect on *T. cruzi* replication, but, crucially, did not interfere with the role played by the immune system in parasite control, reinforcing previous studies (Pereira et al., 2015b; Vilar-Pereira et al., 2016). Furthermore, PTX and Bz+PTX therapies also improved electrical abnormalities as previously shown (Vilar-Pereira et al., 2016), and the addition of PTX to Bz chemotherapy did not interfere with the beneficial effects of Bz controlling parasite load. Crucially, Bz, PTX, and the Bz+PTX combined therapy reduced cardiac fibrosis, which was reproduced in Bz-treated mice in previous studies (Andrade et al., 1991), showing that etiological treatment was effective in regressing inflammatory lesions, including FN deposition, and here, we showed that this is possible using an suboptimal dose of Bz. Altogether, these data support that CCC pathogenesis is complex with contribution of parasite persistence and endogenous host factors, yet to be clarified. Moreover, our findings support a complementary effect of these drugs on the treatment of CCC, opening opportunities to unveil molecular mechanisms and signaling pathways underpinning Chagas heart disease.

Transcriptome profiling have been performed in heart samples of patients (Cunha-Neto et al., 2005; Deng et al., 2013) and mice (Mukherjee et al., 2003; Soares et al., 2010) with CCC, and proteomic studies have been done using collected samples obtained by *in vivo* or *in vitro* experimental studies (Hennig et al., 2019; Nisimura et al., 2020; Wozniak et al., 2020). However, these studies focused on the global expression profiling of the affected heart, and specific immune response gene profiling, specially upon therapy, has not been done to date. Thus, we analyzed the expression of a panel of 92 immune response genes in the heart tissue of chronically infected mice, aiming to identify players of the immune response associated with CCC, exploring the effects of therapeutic strategies in this scenario. Taken together, our immune response gene assessment has revealed that chronic infection caused by experimental *T. cruzi* infection was able to induce a vast change in the immune molecular profile in the affected cardiac tissue. These findings corroborate an intense systemic immune dysregulation shown in several other studies (Machado et al., 2000; Perez-Fuentes, 2003; Pérez et al., 2011; Ferreira et al., 2017; Wozniak et al., 2020), where researchers have found cytokine and chemokine activity to be one of the main altered pathways in the chronic Chagas heart (Soares et al., 2010; Wozniak et al., 2020). Increased IFN-γ expression during CCC was already validated by several studies (Talvani et al., 2000; Abel et al., 2001; Medeiros et al., 2009; Junqueira et al., 2010; Cunha-Neto and Chevillard, 2014; Natale et al., 2018), and functionally, IFN-γ has long been shown to play a pivotal role in parasite replication control and establishment of a Th1-driven immune response (Abrahamsohn and Coffman, 1996; Gomes et al., 2003). In *T. cruzi* infection, treatment of IL-10–deficient mice with anti–IL-12 monoclonal antibody increased parasitemia, showing that IL-12, together with IFN-γ and TNF produced by innate immune cells, plays a pivotal role in controlling the parasitemia in early stages of infection (Abrahamsohn and Coffman, 1996). More recently, high levels of IL-12 have been found in the sera of cardiopathic CD patients (Poveda et al., 2014), and it is known to enhance *T. cruzi* antigen-specific stimulation of peripheral blood mononuclear cells, helping to establish a more oriented and specific immune response, also stimulating IFN-γ and IL-2 production (de Barros-Mazon et al., 1997; Meyer Zum Büschenfelde et al., 1997). Levels of IL-2 were previously found increased in the experimental acute CD (Mateus et al., 2019) and serum of cardiopathic CD patients (Poveda et al., 2014), corroborating our finding for the vehicle-treated group. Moreover, granzyme B (GZMB), secreted by NK cells and cytotoxic T-cells, is a well-known T-cell mechanism in killing intracellular parasites through granulysin, perforin, and granzyme production (Dotiwala et al., 2016). Interestingly, granzyme A^+^ CD8^+^ T lymphocytes were detected in cardiac lesions CD patients (Reis et al., 1993), and patients with advanced CCC show higher proportion of CD8^+^ cytotoxic T cells producing granzyme B and perforin than patients in early stages of the heart disease (Lasso et al., 2015), suggesting a dysfunctionality in T lymphocyte response as disease progresses (Mateus et al., 2019). Indeed, previous study has shown accumulation of perforin^+^ CD8^+^ T cells in the heart tissue associated with progression of cardiomyocyte lesion (Silverio et al., 2012) in the experimental model we used, raising the possibility that the increased cytotoxic activity may involve GZMB activity.

There are no previous studies showing augmented expression for CD3ϵ chain in CD, a chain of the CD3/T-cell receptor complex (TCR) responsible for antigen recognition and T-cell activation, although previous study expression profiling of hearts obtained of transplanted CCC patients found TCR upregulation (Cunha-Neto et al., 2005). These data suggest that the strong Th1-type response we found in the heart tissue of chronically infected mice could be due to CD3/TCR^+^ antigen-specific Th cells response, the most important source of cytokine production found in CCC (Abrahamsohn and Coffman, 1996; Gomes et al., 2003). In the myocardium of CCC patients, increased expression of CCR4 and CCL5 was detected using immunofluorescence (Bilate and Cunha-Neto, 2008), and CCR4 and CXCR3 by RT-qPCR (Cunha-Neto and Chevillard, 2014), corroborating our findings. Further, as expected by the cell populations and the Th1-enriched cytokines found in the heart tissue, the CC and CXC ligands and receptors gene expression have previously been detected in acute and chronically *T. cruzi*–infected mice (Talvani et al., 2000). Moreover, CCR-mediated cell migration has been shown to be crucial for acute and chronic experimental Chagas cardiomyopathy, as CCL5/CCR5- and CCL3-driven cell migration has been found crucial for inflammatory Th1-cell invasion of tissue, cardiac injury, and electrical abnormalities, such as prolonged QTc (Batista et al., 2018; Gibaldi et al., 2020).

Altogether, the upregulation found for these genes in the present study might substantiate the main controlled pathways found for the vehicle-treated group, such as “inflammatory response,” “organismal injury and abnormalities,” “connective tissue disorders,” “cellular development,” “cellular growth and proliferation,” “tissue development,” and “immune cell trafficking” associated with the upregulation of several Th1-driven response molecules, and the lack of regulatory pathways to oppose this strong pro-inflammatory response thereof culminating in tissue remodeling causing the establishment of CCC as extensively shown in previous studies, (Cunha-Neto et al., 2005; Bilate and Cunha-Neto, 2008; Cunha-Neto and Chevillard, 2014), strengthening the molecular findings for our murine CCC model. The proposed molecular signaling pathway, based on our findings, suggests a self-sustaining Th1 immune response marked by the continuous production of the pro-inflammatory cytokine IFN-γ. Despite the limitations on the number of RT-qPCR TaqMan array replicates, our findings here are very well supported by the data in the literature, corroborating an ongoing Th1-driven immune response when the CCC is already installed. These findings are in accordance with previous studies comparing relevant cardiopathy-related microRNAs in CCC and dilated cardiomyopathy where they also found some of these same activated pathways, such as “cardiovascular disease, connective tissue disorders, dermatological diseases” (Ferreira et al., 2014), corroborating a global dysregulation of the immune response caused by *T. cruzi* infection.

Benznidazole treatment regimen for chronic CD has been center of debate in the past few years, as it is associated with numerous adverse effects being an obstacle for patient compliance in endemic areas (Ciapponi et al., 2020). Thus, some clinical trials have been performed or are underway, aiming to reduce Bz dosing or dosing frequency and proposing new treatment protocols, such as MULTIBENZ (Molina-Morant et al., 2020b), BENDITA (Torrico et al., 2021), and TESEO (Almeida et al., 2019). Here, we used a suboptimal dose of Bz (¼ of the usual dose) and our analysis revealed that mice under Bz therapy were able to control the expression of several pivotal genes of immune response. Immunological properties of Bz have been described in other studies (Michailowsky et al., 1998; Sathler-Avelar et al., 2006; Laucella et al., 2009; Ronco et al., 2011; Albareda and Laucella, 2015; Cevey et al., 2016; Vilar-Pereira et al., 2016; Lambertucci et al., 2017); however, in the treatment of CD, specially CCC, the observation of an antiparasitic agent also as an immunomodulatory agent was not yet explored. By killing the parasite subsequently reducing parasite load, the availability of antigen to maintain the *T. cruzi*-specific effector T cells also diminishes, therefore, causing a rearrangement in Th1-driven immunological response contributing to a more regulatory milieu in humans (Albareda and Laucella, 2015). Previous studies aimed to explore the suboptimal dose of Bz alone or in combination with other drugs (Strauss et al., 2013; Khare et al., 2015; Francisco et al., 2016; Hernández et al., 2021; Martins and Sampaio, 2021), and it has been shown that it is effective in decreasing parasite load, attenuating inflammation in the cardiac tissue, and reducing pro-inflammatory cytokines in *T. cruzi*-infected mice and cultured cardiomyocytes (Cevey et al., 2016). Apart from all the modulated immune genes following Bz treatment, another notable finding was the increase in the production of IL-10 by two-fold compared with the vehicle-treated group. Increase in IL-10 production following Bz therapy was previously documented, as CD4^+^ T-cells compartment showed high IL-10 levels in infected children 1 year following Bz treatment (Sathler-Avelar et al., 2008). Furthermore, peripheral blood monocytes obtained from cardiopathic CD patients 1 year after treatment showed an ability to produce regulatory cytokines, such as IL-10, suggesting a shift in immune response balance from a traditional pro-inflammatory response toward a more beneficial and regulatory microenvironment (Campi-Azevedo et al., 2015).

The suboptimal dose of Bz was able to decrease the upregulated IFN-γ, IL-2, CD3ε, and CSF in our validation on individual samples corroborating the findings from the RT-qPCR TaqMan array, which are powerful pro-inflammatory mediators and pivotal contributors for disease progression and CCC onset (Bahia-Oliveira et al., 1998; Cunha-Neto and Chevillard, 2014). Decrease in IFN-γ production has been demonstrated in other studies, in early treatment of *T. cruzi*–infected children, Bz was able to decrease circulating levels of IFN-γ 6 months after therapy discontinuation (Cutrullis et al., 2011), also declining in peripheral IFN-γ-producing T-cells after 12 months, falling below detection levels 36 months after treatment discontinuation of CD patients (Laucella et al., 2009). There is no evidence regarding decrease in IL-2 production after Bz therapy, making this finding unprecedented in our study. In previous studies, Bz treatment did not alter the reported detection of IL-2–producing T-cells responsive to *T. cruzi* in CD patients (Laucella et al., 2009). The expression of CD3 has also not been widely studied in Bz therapy, but it can mean a broad downregulation on activation of cytotoxic T-cells (CD8^+^ naive T-cells) and T helper cells (CD4^+^ naive T-cells) reinforcing that upon elimination of the etiologic agent, i.e., *T. cruzi*, there are less antigens available to trigger immune response (Cunha-Neto and Chevillard, 2014). Furthermore, another main gene that showed regulation upon Bz therapy was CSF2, and studies have not explored yet the production of CSF2 after Bz treatment, but CSF1 when used as a therapy for CCC in mice, induced a mixed Treg/Th1/Th2 immune response that contributed to a persistent cardiac inflammation (González et al., 2013), implying that the role of CSF2 deserves to be further investigated. Crucially, it is important to highlight that even though the Bz therapy was able to regulate the expression of relevant genes, approximately 50 immune-related genes remained dysregulated, corroborating that CD has a strong underlying immune component even in the chronic phase, supporting the lack of efficiency of Bz treatment in some clinical trials carried out in the chronic phase as the BENEFIT trial (Morillo et al., 2015) that did not showed reduction in cardiac clinical deterioration through 5 years of follow-up. Thus, this result supports the need of an associated treatment that could potentially ameliorate the CCC’s cardiac impairment, improving patient’s quality of life.

On the other hand, combined Bz+PTX therapy was also able to modulate relevant genes, and this therapeutic strategy revealed two additional controlled pathways related to “cell death and survival” and “organismal survival” mostly related to the regulation of the PRF1 gene. In addition to PTX traditional mechanism of action on increasing blood flow improving its rheological properties, PTX also has anti-inflammatory and antioxidant effects (Pereira et al., 2015b; McCarty et al., 2016; Vilar-Pereira et al., 2016). PTX can inhibit a wide range of cytotoxic responses, either in a directly, decreasing expression of perforin/granzymes, or indirectly manner, acting on cytokine production dynamics, which helps to explain the results we have here (Shaw et al., 2009). Indeed, PTX-treated chronically infected mice has reduced numbers of perforin^+^ cells infiltrating the heart tissue (Pereira et al., 2015b). PTX therapy has shown attenuation of cell surface expression of IL-2 receptor in human lymphocytes (Rao et al., 1991), but its effect on IL-2 cytokine expression has not been shown to date, especially in CD. Furthermore, PTX also contributed to downregulation of GM-CSF, TNF, and IFN-γ in CD8^+^ HIV-specific cytotoxic T lymphocytes (Heinkelein et al., 1995). C3, a key protein in the complement system, had allotypes related to the susceptibility to CD and the development of cardiomyopathy (Messias-Reason et al., 2003), suggesting that PTX might act in the complement signaling pathway and reversion of C3 expression might slow down CCC progression, a matter to be further investigated. Chronic CD patients display deterioration of T-cell function, exhaustion caused by low production of cytokines, and augmented expression/co-expression of inhibitory receptors, such as programmed cell death-1 (PD-1), CTLA-4/CD152, T-cell Ig mucin-3 (TIM-3), CD160, and CD244 (2B4) (Lasso et al., 2015). Our group has evaluated the immunomodulatory properties of PTX in the progression of CCC in mice, showing a repositioned CD8^+^ T-cell response toward homeostasis, which corroborates our finding of Bz+PTX acting on the T-cell exhaustion pathway (Pereira et al., 2015b; Vilar-Pereira et al., 2016).

Among the study main limitations, we include the lack of the echocardiogram exam, as was done in previous studies using the same model (Pereira et al., 2014a; Pereira et al., 2015b), for evaluation of cardiac cavities and LVEF (Gibaldi et al., 2020). CK-MB levels for characterization of cardiac damage was also not evaluated; however, the establishment of the cardiomyopathy was very well characterized through electrocardiogram, parasite load, and fibronectin assessment. There is also the limitation of only one replicate per TaqMan array, done with a pool of total RNA of three different mice from each experimental group. The TaqMan array was used as a global assessment of the immune response genes to orientate the selection of genes for further validation in individual samples under therapy, which was done to validate further selected genes of interest.

Overall, it is important to highlight that therapy with suboptimal dose of Bz brings back the discussion of dose optimization and, moreover, showed exceptional immunomodulatory properties of Bz in the cardiac tissue. Thus, this result reinforces the need of etiological therapy to promote *T. cruzi* control, and consequently decrease the availability of antigens, contributing to repositioning of the immune response. Although Bz therapy had impacted, only the combined Bz+PTX therapy ameliorated all analyzed electrical abnormalities, hampering the progression of CCC. In summary, our findings reinforce that parasite persistence and dysregulated immune response underpin CCC severity, and therefore, Bz and Bz+PTX emerge as rational tools to interfere in these targets, aiming to improve CCC prognosis in chronic CD.

## Data Availability Statement

The raw data supporting the conclusions of this article will be made available by the authors, without undue reservation.

## Ethics Statement

The Institutional Committee for Animal Ethics of Fiocruz (CEUA-Fiocruz L004/09 and LW10/14) approved all experimental procedures used in the present study.

## Author Contributions

PF performed the majority of the experiments, analyzed data sets, and wrote the manuscript. GV-P and IP contributed to the *in vivo* experimental model of CCC. SR and KB performed the network pathway analysis and IA contributed to the data set and manuscript analysis. OM and JL-V were responsible for experimental design and data analysis. OM was responsible for the final manuscript revision. All authors contributed to the article and approved the submitted version.

## Funding

This study was financed in part by the Coordination for the Improvement of Higher Education Personnel (Coordenação de Aperfeiçoamento de Pessoal de Nível Superior - CAPES) - Finance Code 001. This work also received financial support from Conselho Nacional de Desenvolvimento Científico e Tecnológico (CNPq), Fundação Carlos Chagas Filho de Amparo à Pesquisa do Rio de Janeiro (FAPERJ). OM is a researcher fellow of CNPq1D (311539/2020-3) and FAPERJ (JCNE, E-26/203.031/2018). JL-V is fellow of CNPq1B (BPP 306037/2019-0) and FAPERJ (CNE, E-26/210.190/2018). IA partially supported by grant 5U54MD007592 from the National Institute on Minority Health and Health Disparities (NIMHD), a component of the National Institutes of Health (NIH).

## Conflict of Interest

The authors declare that the research was conducted in the absence of any commercial or financial relationships that could be construed as a potential conflict of interest.
