# Supplementary material for: Treatment With Suboptimal Dose of Benznidazole Mitigates Immune Response Molecular Pathways in Mice With Chronic Chagas Cardiomyopathy
Source: Front Cell Infect Microbiol. 2021 Jul 14;11:692655. doi: 10.3389/fcimb.2021.692655 (PMC8351877; doi:10.3389/fcimb.2021.692655)
Supplement: Supplementary file 1 [file DataSheet_1.docx]

**Supplementary Material**

**Suplemmentary table 1**. List of genes expressed in at least 2.0 fold change

|  |  | Fold change | | |
| --- | --- | --- | --- | --- |
| Name | **Acession Number** | **Infected** | **Bz** | **Bz+PTX** |
| IFNG | NM_008337.3 | 126.5070038 | 11.15999985 | 8.708999634 |
| GZMB | NM_013542.2 | 70.35900116 | 39.69599915 | 32.14799881 |
| CD3ε | NM_007648.4 | 65.61000061 | 7.793000221 | 7.109000206 |
| IL-12b | AF128214.1 | 44.34799957 | 9.211999893 | 5.238999844 |
| CCR4 | NM_009916.2 | 36.06000137 | 4.722000122 | 6.34499979 |
| CCL3 | NM_011337.2 | 23.46199989 | 7.762000084 | 5.472000122 |
| CD40lg | NM_011616.2 | 23.18199921 | 6.236999989 | 4.666999817 |
| CD8a | NM_001081110.2 | 22.66300011 | 14.89599991 | 14.67599964 |
| IL-2 | NM_008366.3 | 18.20199966 | 2.22300005 | 2.680999994 |
| IL-10 | NM_010548.2 | 16.56599998 | 29.69400024 | 4.138000011 |
| CCL5 | NM_013653.3 | 15.74300003 | 23.70199966 | 9.416000366 |
| CSF2 | NM_009969.4 | 15.65499973 | 1.636000037 | 1.271000028 |
| TBX21 | NM_019507.2 | 10.66300011 | 7.254000187 | 6.948999882 |
| FasL | NM_001205243.1 | 9.93200016 | 3.631000042 | 7.644999981 |
| CXCL10 | Mm00445235_m1 | 7.977000237 | 10.5710001 | 4.18200016 |
| CXCR3 | AB003174.1 | 7.772999763 | 7.230000019 | 5.046999931 |
| CD4 | NM_013488.2 | 7.394999981 | 6.504000187 | 5.65199995 |
| CXCL11 | NM_019494.1 | 5.813000202 | 7.953000069 | 1.731999993 |
| IL-6 | NM_031168.1 | 3.884000063 | 0.232999995 | 0.172000006 |
| PRF1 | NM_011073.3 | 3.532999992 | 4.206999779 | 1.902999997 |
| H2-EB1 | NM_010382.2 | 3.447000027 | 2.059999943 | 2.063999891 |
| TNFRSF18 | NM_009400.2 | 3.322000027 | 4.769999981 | 3.088000059 |
| STAT4 | AK132675.1 | 2.819999933 | 2.950999975 | 1.904999971 |
| CD80 | NM_009855.2 | 2.733999968 | 0.527999997 | 0.43599999 |
| STAT1 | NM_001205313.1 | 2.528000116 | 4.504000187 | 1.080000043 |
| CD28 | NM_007642.4 | 2.345000029 | 2.210000038 | 1.110999942 |
| CD40 | NM_011611.2 | 2.338000059 | 2.029000044 | 0.671000004 |
| IL-7 | NM_008371.4 | 2.242000103 | 0.938000023 | 0.666000009 |
| C3 | BC043338.1 | 2.157000065 | 1.008999944 | 0.779999971 |
| IL-12a | AF128210.1 | 2.124000072 | 0.934000015 | 1.473999977 |
| SELP | NM_011347.2 | 0.393000007 | 0.356000006 | 0.351000011 |
| SOCS1 | NM_001271603.1 | 0.388000011 | 0.333000004 | 0.439999998 |
| Fas | NM_001146708.1 | 0.351999998 | 0.495999992 | 0.405000001 |
| CSF1 | NM_001113529.1 | 0.335999995 | 0.449999988 | 0.414999992 |
| LIF | NM_008501.2 | 0.326999992 | 0.467000008 | 0.286000013 |
| SOCS2 | NM_001168655.1 | 0.321999997 | 0.163000003 | 0.137999997 |
| IKBKB | NM_001159774.1 | 0.296999991 | 0.254999995 | 0.270999998 |
| SMAD3 | NM_016769.4 | 0.273000002 | 0.319999993 | 0.257999986 |
| NFKB1 | NM_008689.2 | 0.257999986 | 0.493000001 | 0.300999999 |
| TGFB1 | NM_011577.1 | 0.240999997 | 0.170000002 | 0.067000002 |
| CD19 | NM_009844.2 | 0.237000003 | 0.703000009 | 0.972000003 |
| STAT6 | NM_009284.2 | 0.237000003 | 0.930000007 | 0.42899999 |
| IL-2RA | NM_008367.3 | 0.224999994 | 0.477999985 | 0.51700002 |
| SELE | NM_011345.2 | 0.224999994 | 1.18599999 | 0.545000017 |
| ACE | NM_001281819.1 | 0.218999997 | 0.629000008 | 0.44600001 |
| SKI | NM_011385.2 | 0.216000006 | 0.291000009 | 0.208000004 |
| ICAM1 | NM_010493.2 | 0.215000004 | 0.407999992 | 0.228 |
| NFKB2 | NM_001177369.1 | 0.210999995 | 0.462000012 | 0.356000006 |
| STAT3 | NM_011486.4 | 0.185000002 | 0.611999989 | 0.395000011 |
| BCL2 | NM_009741.4 | 0.166999996 | 0.555000007 | 0.36500001 |
| HMOX1 | NM_010442.2 | 0.163000003 | 0.611999989 | 0.444000006 |
| EDN1 | NM_010104.3 | 0.140000001 | 0.319000006 | 0.323000014 |
| FN1 | NM_001276408.1 | 0.133000001 | 0.150000006 | 0.301999986 |
| NFATC3 | NM_010901.2 | 0.104000002 | 0.291999996 | 0.328999996 |
| NOS2 | NM_010927.3 | 0.100000001 | 0.407999992 | 0.328000009 |
| CD34 | NM_001111059.1 | 0.094999999 | 0.601000011 | 0.367000014 |
| BAX | NM_007527.3 | 0.090999998 | 0.432000011 | 0.344999999 |
| VEGFA | NM_001025250.3 | 0.067000002 | 0.32100001 | 0.326999992 |
| BCL2l1 | NM_001289716.1 | 0.045000002 | 0.136000007 | 0.231999993 |
| IL-15 | NM_001254747.1 | 0.043000001 | 0.421000004 | 0.411000013 |
| LRP2 | NM_001081088.1 | 0.028000001 | 0.023 | 0.337000012 |

**Suplemmentary table 2.** List of genes with expression restored with Bz treatment

|  |  | Fold change | | |
| --- | --- | --- | --- | --- |
| Name | **Acession Number** | **Infected** | **Bz** | **Bz+PTX** |
| CSF2 | NM_009969.4 | 15.65499973 | 1.636000037 | 1.271000028 |
| CD80 | NM_009855.2 | 2.733999968 | 0.527999997 | 0.43599999 |
| IL-7 | NM_008371.4 | 2.242000103 | 0.938000023 | 0.666000009 |
| C3 | BC043338.1 | 2.157000065 | 1.008999944 | 0.779999971 |
| IL-12a | AF128210.1 | 2.124000072 | 0.934000015 | 1.473999977 |
| STAT6 | NM_009284.2 | 0.237000003 | 0.930000007 | 0.42899999 |
| CD19 | NM_009844.2 | 0.237000003 | 0.703000009 | 0.972000003 |
| SELE | NM_011345.2 | 0.224999994 | 1.18599999 | 0.545000017 |
| ACE | NM_001281819.1 | 0.218999997 | 0.629000008 | 0.44600001 |
| STAT3 | NM_011486.4 | 0.185000002 | 0.611999989 | 0.395000011 |
| BCL2 | NM_009741.4 | 0.166999996 | 0.555000007 | 0.36500001 |
| HMOX1 | NM_010442.2 | 0.163000003 | 0.611999989 | 0.444000006 |
| CD34 | NM_001111059.1 | 0.094999999 | 0.601000011 | 0.367000014 |

**Suplemmentary table 3.** List of genes with expression restored with Bz+PTX treatment

|  |  | Fold change | | |
| --- | --- | --- | --- | --- |
| Name | **Acession Number** | **Infected** | **Bz** | **Bz+PTX** |
| CSF2 | NM_009969.4 | 15.65499973 | 1.636000037 | 1.271000028 |
| CXCL11 | NM_019494.1 | 5.813000202 | 7.953000069 | 1.731999993 |
| PRF1 | NM_011073.3 | 3.532999992 | 4.206999779 | 1.902999997 |
| STAT4 | AK132675.1 | 2.819999933 | 2.950999975 | 1.904999971 |
| STAT1 | NM_001205313.1 | 2.528000116 | 4.504000187 | 1.080000043 |
| CD28 | NM_007642.4 | 2.345000029 | 2.210000038 | 1.110999942 |
| CD40 | NM_011611.2 | 2.338000059 | 2.029000044 | 0.671000004 |
| IL-7 | NM_008371.4 | 2.242000103 | 0.938000023 | 0.666000009 |
| C3 | BC043338.1 | 2.157000065 | 1.008999944 | 0.779999971 |
| IL-12a | AF128210.1 | 2.124000072 | 0.934000015 | 1.473999977 |
| CD19 | NM_009844.2 | 0.237000003 | 0.703000009 | 0.972000003 |
| IL-2RA | NM_008367.3 | 0.224999994 | 0.477999985 | 0.51700002 |
| SELE | NM_011345.2 | 0.224999994 | 1.18599999 | 0.545000017 |

**
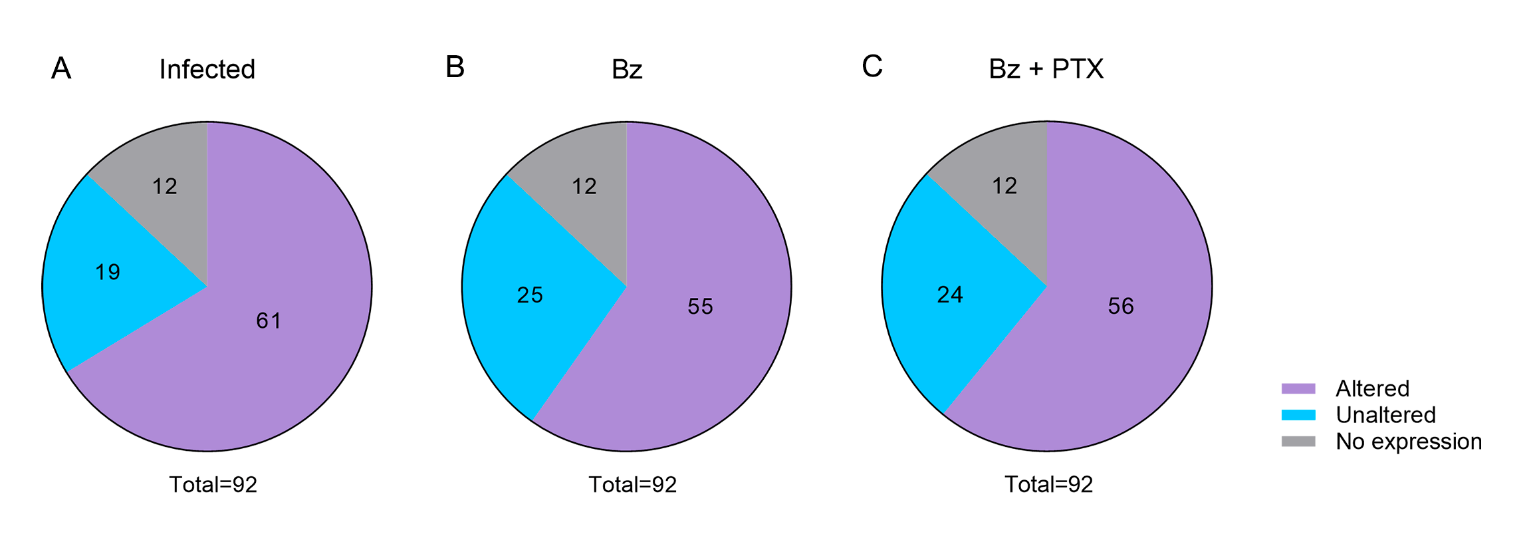
**

**Supplementary Figure 1.** Overview of number of genes altered/unaltered in (A) infected, (B) Bz and (C) Bz+PTX groups.

**
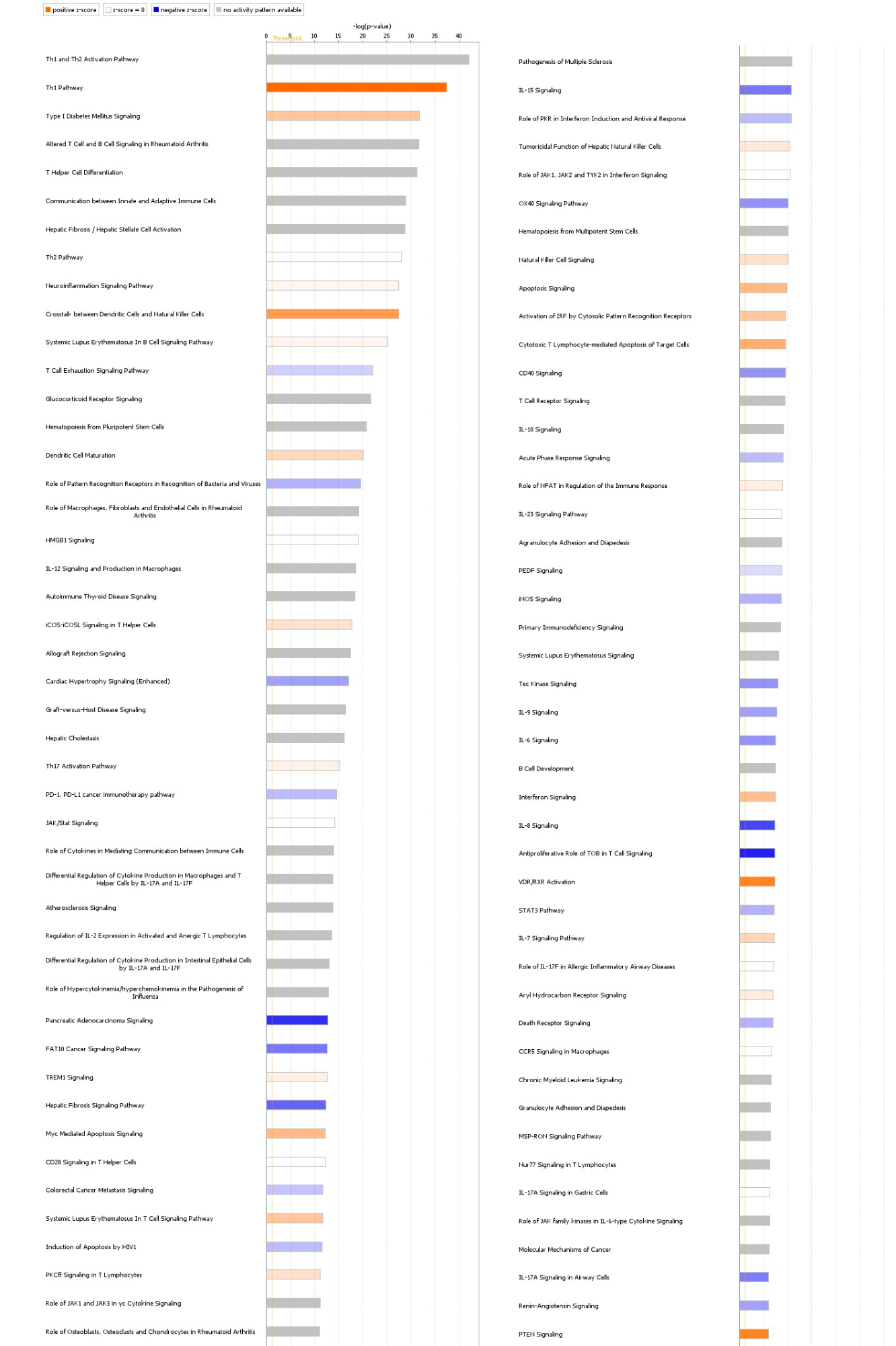
Supplementary Figure 2****.** Top canonical pathways activated (shades of orange) or inhibited (shades of blues) by the altered immune response genes in the infected group.

**
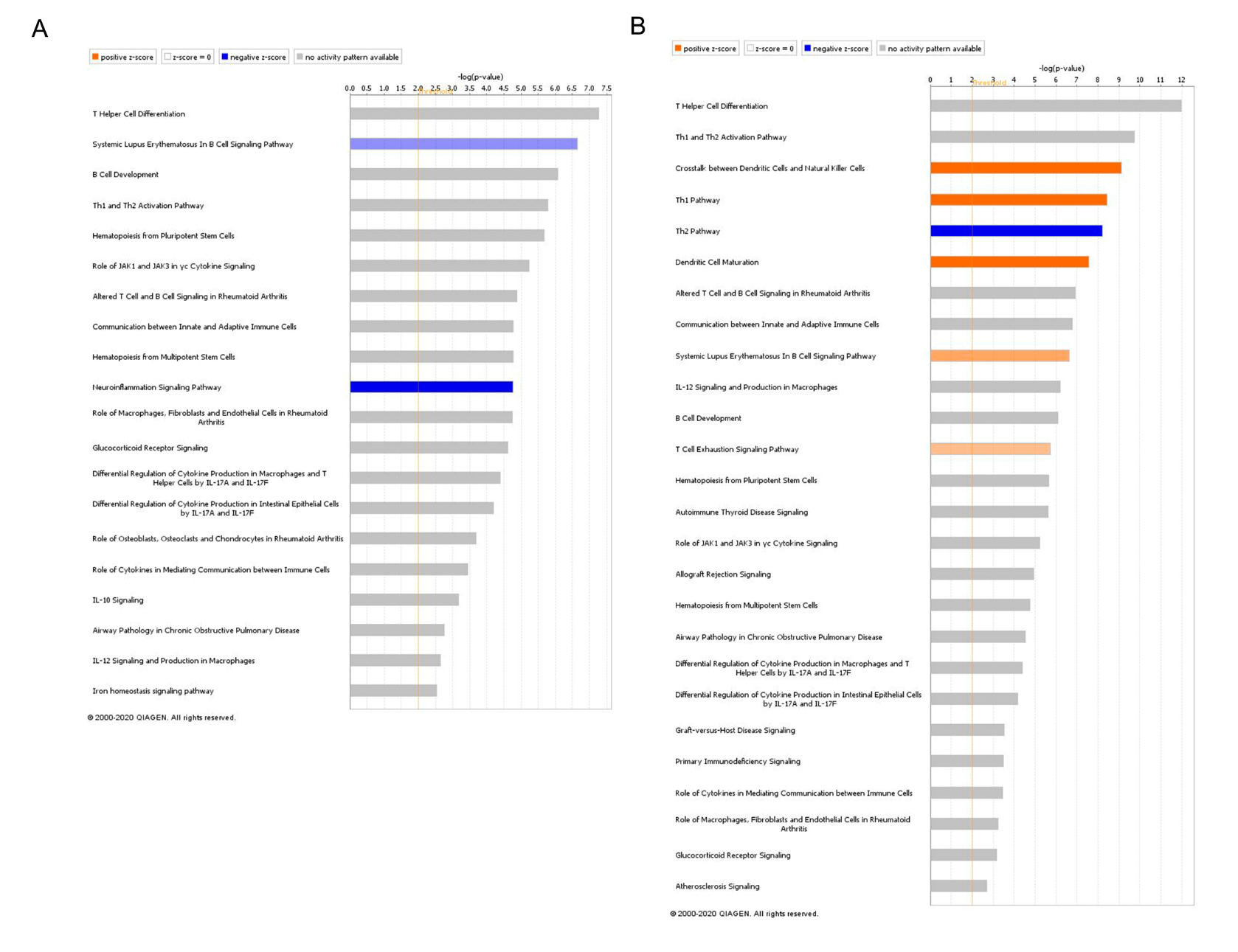
**

**Supplementary Figure 3.** Top canonical pathways activated (shades of orange) or inhibited (shades of blues) by the altered immune response genes in the (A) Bz and (B) Bz+ PTX group.

**
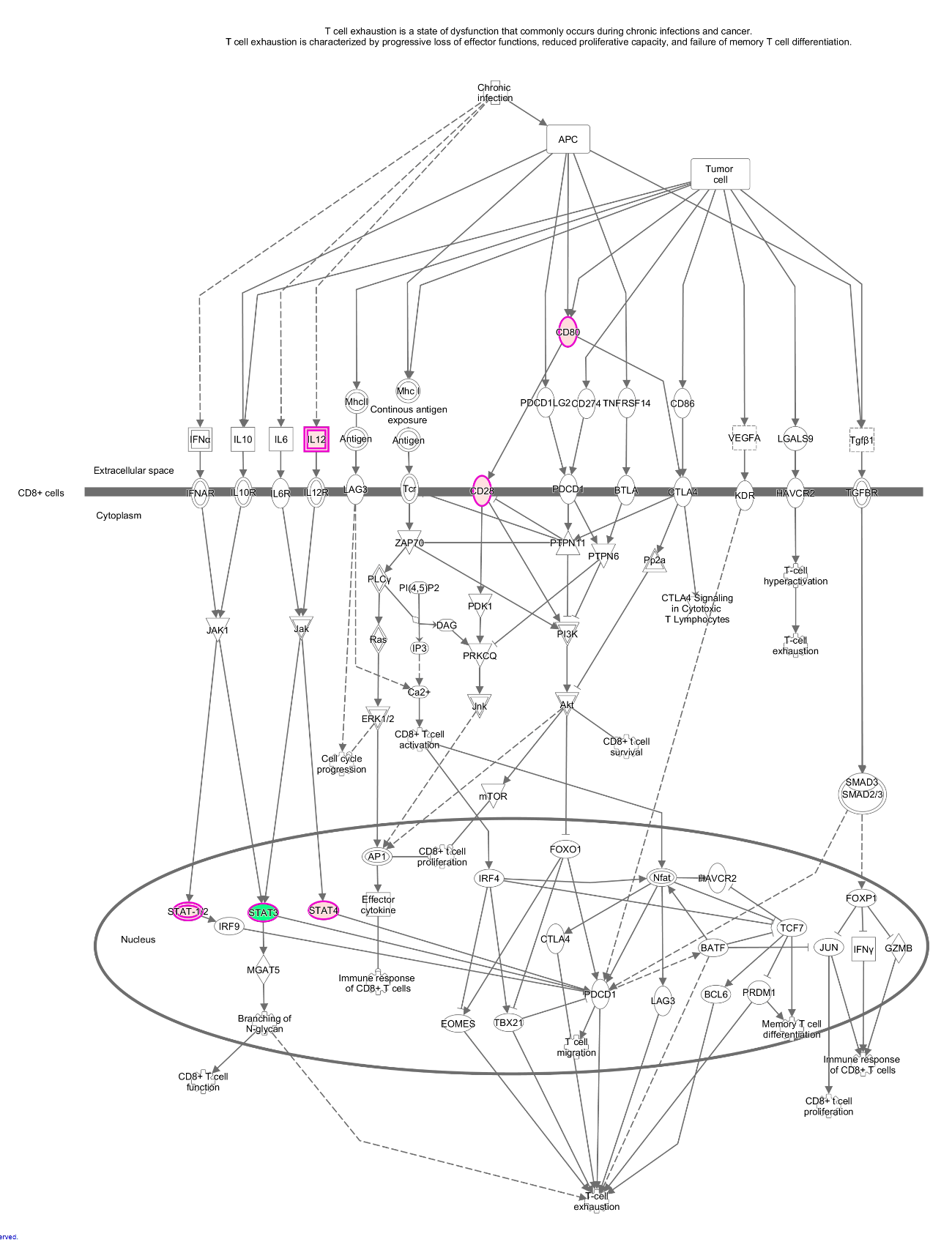
**

**Supplementary Figure 4.** T cell exhaustion pathway representation. In silico analysis done using IPA software (QIAGEN, USA) showing a signaling pathway built with: IL-12, CD80, CD28, STAT1/2, STAT4. The molecules are highlighted with fuschia/pink outlines and filled with different shades of red or green color which indicate up and down-regulation, respectively, based on their fold change values from the infected group compared to the non-infected group. Direct line: direct activation; dashed line: indirect activation.
